# Characterizing hedgehog pathway features in senescence associated osteoarthritis through Integrative multi-omics and machine learning analysis

**DOI:** 10.3389/fgene.2024.1255455

**Published:** 2024-02-20

**Authors:** Tao Wang, Zhengrui Li, Shijian Zhao, Ying Liu, Wenliang Guo, Raquel Alarcòn Rodrìguez, Yinteng Wu, Ruqiong Wei

**Affiliations:** ^1^ Department of Orthopedic Joint, The First Affiliated Hospital of Guangxi Medical University, Nanning, Guangxi, China; ^2^ Shanghai Jiao Tong University School of Medicine, Shanghai, China; ^3^ Department of Cardiology, The Affiliated Cardiovascular Hospital of Kunming Medical University (Fuwai Yunnan Cardiovascular Hospital), Kunming, Yunnan, China; ^4^ Department of Rehabilitation Medicine, The First Affiliated Hospital of Guangxi Medical University, Nanning, Guangxi, China; ^5^ Department of Rehabilitation Medicine, The Eighth Affiliated Hospital of Guangxi Medical University, Guigang, Guangxi, China; ^6^ Faculty of Health Sciences, University of Almerìa, Almeria, Spain; ^7^ Department of Orthopedic and Trauma Surgery, the First Affiliated Hospital of Guangxi Medical University, Nanning, Guangxi, China

**Keywords:** hedgehog, osteoarthritis (OA), senescence, bulk RNA sequencing (bulk-RNA seq), single-cell RNA sequencing (scRNA-seq), immune cell infiltration, molecular clusters

## Abstract

**Purpose:** Osteoarthritis (OA) is a disease of senescence and inflammation. Hedgehog’s role in OA mechanisms is unclear. This study combines Bulk RNA-seq and scRNA-seq to identify Hedgehog-associated genes in OA, investigating their impact on the pathogenesis of OA.

**Materials and methods:** Download and merge eight bulk-RNA seq datasets from GEO, also obtain a scRNA-seq dataset for validation and analysis. Analyze Hedgehog pathway activity in OA using bulk-RNA seq datasets. Use ten machine learning algorithms to identify important Hedgehog-associated genes, validate predictive models. Perform GSEA to investigate functional implications of identified Hedgehog-associated genes. Assess immune infiltration in OA using Cibersort and MCP-counter algorithms. Utilize ConsensusClusterPlus package to identify Hedgehog-related subgroups. Conduct WGCNA to identify key modules enriched based on Hedgehog-related subgroups. Characterization of genes by methylation and GWAS analysis. Evaluate Hedgehog pathway activity, expression of hub genes, pseudotime, and cell communication, in OA chondrocytes using scRNA-seq dataset. Validate Hedgehog-associated gene expression levels through Real-time PCR analysis.

**Results:** The activity of the Hedgehog pathway is significantly enhanced in OA. Additionally, nine important Hedgehog-associated genes have been identified, and the predictive models built using these genes demonstrate strong predictive capabilities. GSEA analysis indicates a significant positive correlation between all seven important Hedgehog-associated genes and lysosomes. Consensus clustering reveals the presence of two hedgehog-related subgroups. In Cluster 1, Hedgehog pathway activity is significantly upregulated and associated with inflammatory pathways. WGCNA identifies that genes in the blue module are most significantly correlated with Cluster 1 and Cluster 2, as well as being involved in extracellular matrix and collagen-related pathways. Single-cell analysis confirms the significant upregulation of the Hedgehog pathway in OA, along with expression changes observed in 5 genes during putative temporal progression. Cell communication analysis suggests an association between low-scoring chondrocytes and macrophages.

**Conclusion:** The Hedgehog pathway is significantly activated in OA and is associated with the extracellular matrix and collagen proteins. It plays a role in regulating immune cells and immune responses.

## 1 Introduction

Osteoarthritis (OA) is a common degenerative joint disorder observed in the field of orthopedics. It is characterized by cartilage degeneration, abnormal bone growth, formation of osteophytes, and thickening beneath the cartilage (Coryell et al., 2021). Pathologically, it involves the hypertrophic differentiation of chondrocytes and changes in the composition of the extracellular matrix (ECM). Well-established risk factors include aging, prior joint injuries, excessive body weight, genetic predisposition, gender, as well as anatomical factors related to joint shape and alignment (Shen et al., 2019). Moreover, severe synovitis exacerbates cartilage erosion, serving as both a risk factor for OA and a common pathological alteration associated with this condition (Xie et al., 2020). OA can induce various symptoms, including joint tenderness, stiffness, and pain, significantly impacting activity levels, physical functioning, sleep quality, and emotional wellbeing. In 2013, the economic burden caused by this disease amounted to approximately $27 billion in the United States, with an alarming loss of labor productivity (Coryell et al., 2021). Presently, clinical diagnosis of OA heavily relies on patients’ clinical manifestations and imaging techniques, leading to the limitations in achieving precise early-stage diagnosis (Zhang et al., 2018). Due to the inability to diagnose OA at an early stage, most patients experience a progression of their condition, resulting in a poorer prognosis and rendering many treatment approaches ineffective. Therefore, further exploration of the pathogenic mechanisms underlying OA is of paramount importance in improving the prognosis for individuals affected by this condition.

In 1980, Nüsslein-Volhard and Wieschaus made the initial discovery of the Hedgehog signaling pathway within the organism *Drosophila melanogaster* (Ingham et al., 2011). It plays a crucial role in mammalian embryonic development, as well as in the growth and differentiation of cells following embryogenesis (Jia et al., 2015). The Hedgehog protein family includes Indian Hedgehog (Ihh)、Sonic Hedgehog (Shh) and Desert Hedgehog (Dhh) (Zhou et al., 2014). GLI1, GLI2, and GLI3 are key downstream effectors of the Hh signaling pathway, acting as nuclear transcription factors that bind to promoters to regulate target gene expression (Briscoe and Thérond, 2013). This pathway plays a crucial role in embryonic development by controlling growth through the regulation of chondrocyte development and promoting endochondral ossification. During the process of endochondral bone growth, prehypertrophic chondrocytes are primarily responsible for producing and secreting a substance that plays a crucial role in the proliferation and differentiation of these cells (Hsu et al., 2011). The Hh signaling pathway can stimulate the maturation and mineralization of chondrocytes (Bechtold et al., 2016). Ihh is primarily produced and secreted by prehypertrophic chondrocytes and regulates chondrocyte hypertrophy and endochondral ossification during growth plate development. Transgenic mice with induced Ihh expression exhibit chondrocyte hypertrophy and cartilage damage similar to human osteoarthritis (Zhou et al., 2014). A study has shown that the Hedgehog signaling pathway regulates cholesterol homeostasis in chondrocytes. The accumulation of cholesterol within these cells is associated with increased disease severity. The researchers observed a positive correlation between elevated levels of Gli-mediated transcription in chondrocytes and intracellular cholesterol accumulation as well as disease severity (Ali et al., 2016). In OA, activation of the Hh pathway typically induces upregulation of hypertrophic markers, including type X collagen, increased production of nitric oxide and prostaglandin E2, as well as the secretion of various matrix-degrading enzymes. These changes ultimately lead to cartilage degeneration and contribute to the development of OA (Xiao et al., 2020). The current research on the Hedgehog signaling pathway in OA is limited. However, given its potential role as a crucial component in the pathogenesis and progression of OA-related changes in bone joints, targeting this pathway could offer a promising approach for preventing or treating OA. Therefore, it is imperative to investigate the molecular mechanisms underlying the Hedgehog signaling pathway in the context of OA.

**FIGURE 1:**
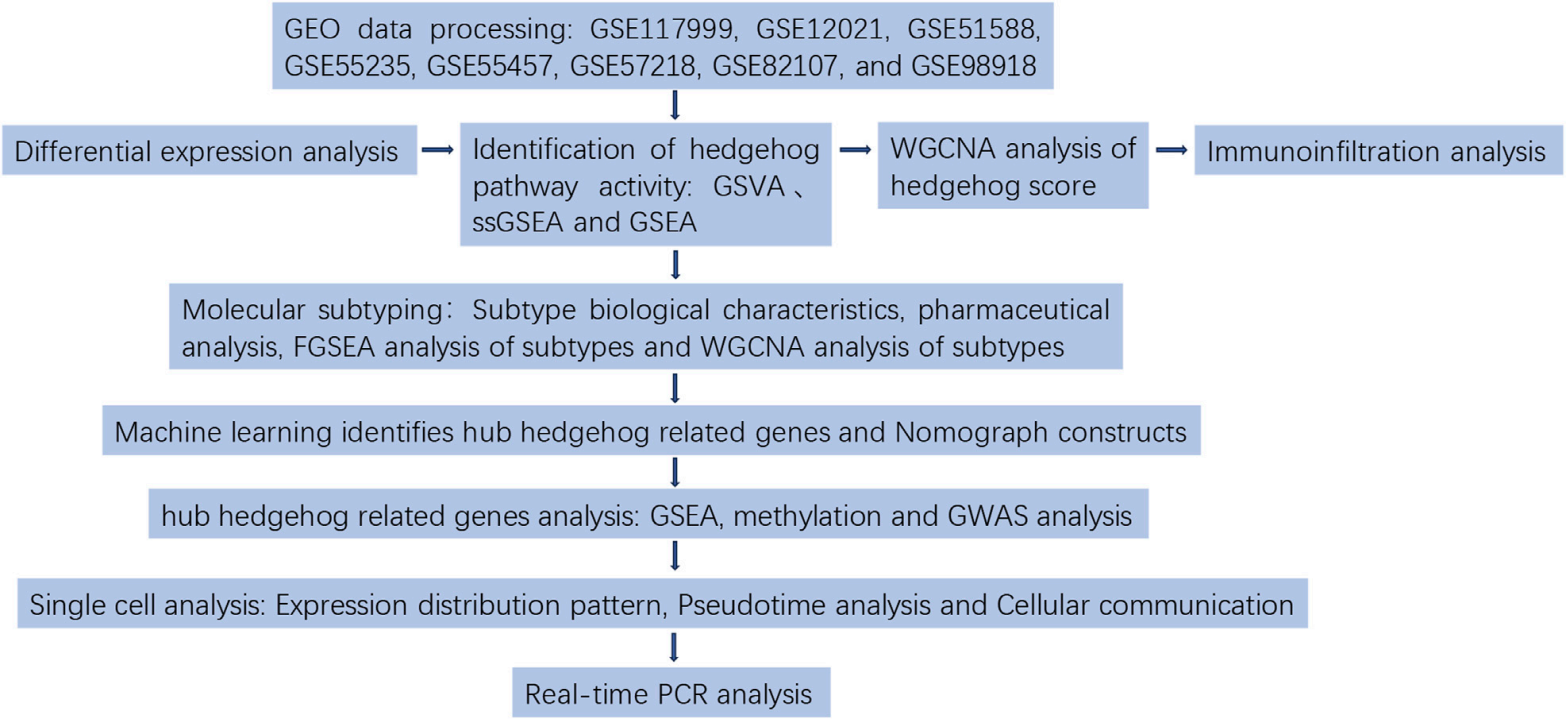
Flow chart.

In this study, we employed single-sample Gene Set Enrichment Analysis (ssGSEA), Gene Set Variation Analysis (GSVA), and GSEA to assess the activity of the Hedgehog signaling pathway. Weighted Gene Co-expression Network Analysis (WGCNA) was utilized to identify modules associated with OA and Hedgehog score for subsequent enrichment analysis. Additionally, ten machine learning methods, including lasso regression, ridge regression, elastic net regression, SVM-RFE, random forest, bagging, GBM, XGBoost-xgbTree, XGBoost-xgbLinear, and decision tree were applied to identify hub genes related to Hedgehog and construct prediction models to evaluate their reliability. Mechanistic insights into hub Hedgehog-related genes were further explored through GSEA analysis. After analyzing the expression profiles of Hedgehog-related genes, we categorized 135 patients with OA into two subgroups based on their Hedgehog-related characteristics. We subsequently investigated the Hedgehog activity, immune profile, drugs, functions, and pathways between these two clusters. Their genomes were analyzed using methylation and GWAS. Single-cell analysis was conducted to examine the expression patterns, pseudotemporal changes, cell communication, and transcription factors related to hub Hedgehog genes. Finally, we conducted Real-time PCR analysis to confirm the expression levels of key Hedgehog-related genes. The process of this study is shown in Figure 1.

## 2 Materials and methods

### 2.1 Data acquisition and preprocessing

The OA-related microarray datasets, including GSE117999, GSE12021, GSE51588, GSE55235, GSE55457, GSE57218, GSE82107, and GSE98918, should be downloaded from the GEO database (https://www.ncbi.nlm.nih.gov/geo/). Additionally, retrieve the OA-related single-cell sequencing dataset, GSE133449, from the GEO database. For processing the microarray datasets, match the probe and gene names based on the provided annotation information for each GPL platform. When multiple probes correspond to the same gene, select the probe with the highest expression level to maintain consistency. Normalize the expression matrix using the normalizeBetweenArrays function and perform log2 transformation on datasets requiring it. Retrieve the common genes across all eight datasets after their integration. To normalize the expression values across different batches or platforms, utilize the ComBat method from the “sva” R package. Assess the removal of batch effects through principal component analysis.

### 2.2 Differential expression analysis

To initiate the analysis, the hedgehog gene set can be accessed from the MSigDB database through the following link: https://www.gsea-msigdb.org/gsea. Subsequently, the “limma” R package can be utilized to assess the differentially expressed genes (DEGs) between samples with osteoarthritis (OA) and normal samples. The filtering criterion for identifying hedgehog-related DEGs is a corrected *p*-value <0.05. In order to visually represent the expression levels within each sample, a heatmap showcasing the hedgehog gene set can be created. Additionally, a volcano plot can be generated to visualize the differential expression of hedgehog-related DEGs. Furthermore, the “wilcox.test” algorithm can be employed to analyze the differential expression levels of the hedgehog gene set in OA and normal samples. Moreover, it is important to investigate the correlation of hedgehog-related DEGs using this analysis.

### 2.3 Enrichment analysis

The GSVA method is a valuable technique utilized for the analysis of gene expression data (Hänzelmann et al., 2013). It allows for the assessment of the overall activity level of gene sets within each biological sample. By employing the “GSVA” R package, it is possible to map the gene expression data of each sample to the hedgehog gene set and calculate their respective activity levels. On the other hand, ssGSEA takes into account not only the expression status of genes within a gene set but also considers the expression levels of individual genes. Utilizing the ssGSEA algorithm, the gene expression data of each sample can be mapped to the hedgehog gene set, enabling the computation of enrichment scores that reflect the overall activity level of the gene set within that specific sample. To establish a comprehensive gene co-expression network, the Weighted Gene Co-expression Network Analysis (WGCNA) approach will be employed (Langfelder and Horvath, 2008), utilizing the top 5,000 highly variable genes, grouping information, and GSVA scores of the hedgehog gene set as input variables. This network analysis will help identify modules within the network and uncover key genes that are associated with the provided input variables. Further analysis will involve conducting Gene Ontology (GO) (Gene Ontology Consortium, 2006) and Kyoto Encyclopedia of Genes and Genomes (KEGG) (Kanehisa and Goto, 2000) analyses on the key module genes using the “clusterProfiler” R package. These analyses will provide insights into the functional annotations and pathways associated with the identified key module genes.

### 2.4 Immuno-infiltration analysis

To assess the relative abundance of different immune cell types, a combination of the CIBERSORT and MCP-counter algorithms can be employed (Newman et al., 2015; Becht et al., 2016). These algorithms will enable the calculation of the relative abundances of immune cell types. The resulting immune cell abundances can be visualized using a cumulative histogram. To analyze the differential expression levels of immune cells between OA and control samples, the wilcox. test algorithm can be utilized. It is important to exclude the gene expression data and immune cell abundance from control samples during this analysis.

To investigate the correlation between differentially expressed genes (DEGs) related to the hedgehog pathway and immune cells, the Spearman algorithm can be used. This analysis will provide insights into the association between hedgehog-related DEGs and specific immune cell populations.

### 2.5 Consensus clustering

To classify OA cases into subgroups, consensus clustering was performed. In this procedure, the K-means algorithm with the Euclidean distance metric was utilized. The maximum number of clusters was set as 10. The final determination of the cluster number was based on the consensus matrix and the cluster consensus score, which required a score higher than 0.8 to ensure robustness and reliability in the clustering results.

### 2.6 Characterization of subpopulations

Using the hallmark gene set as the background gene set, we applied the GSVA method to investigate pathway differences among the subgroups. This analysis allowed us to identify and compare the activity levels of different pathways across the subgroups. Furthermore, we employed ssGSEA to specifically explore pathway differences related to hedgehog signaling between the subgroups. By focusing on the hedgehog pathway, we aimed to understand any variations in its activity among the subgroups. Moreover, we conducted an analysis of the immune characteristics within the subgroups. This involved examining the relative abundance and activity of immune cell types, as well as any potential associations with the subgroup classification. Lastly, we evaluated the expression patterns of hedgehog pathway genes within each subgroup. This analysis provided insights into the differential regulation and potential involvement of the hedgehog pathway in driving the observed subgroup distinctions.

### 2.7 Drug analysis

To identify potential drugs suitable for the subtypes, we utilized the differential expression analysis results of the subtypes as the disease signature and accessed the drug information available in the CMap database (https://clue.io) as the drug signature. We employed the eXtreme Sum (XSum) method as the feature matching algorithm to screen for drugs that show similarity or inverse correlation with the disease signature of the subtypes. This approach helped us identify candidate drugs that may have therapeutic potential for the specific subtypes under investigation.

### 2.8 Subtype of FGSEA analysis

To perform differential expression analysis on subtype 1 and subtype 2, we can utilize the “limma” R package. This package provides a robust framework for identifying genes that are differentially expressed between these two subtypes. After conducting the analysis, we can sort the genes based on their log-fold change (logFC) values. To assess whether specific gene sets are enriched in the samples, we can employ the “fgsea” R package for enrichment analysis. This analysis will help us determine if any predefined gene sets show significant enrichment in either subtype. We can set criteria such as an absolute normalized enrichment score (NES) greater than 1 and a *p*-value below 0.01 to filter for pathways that are considered statistically significant. By applying these methods, we can gain insights into the differentially expressed genes and enriched pathways associated with subtype 1 and subtype 2, providing a better understanding of the molecular characteristics and potential functional differences between the two subtypes.

### 2.9 Subtype of WGCNA

To explore the biological functions of each osteoarthritis (OA) subgroup, we employed the Weighted Gene Co-expression Network Analysis (WGCNA) based on their respective characteristics. Initially, we identified the top 5,000 highly variable genes within the subgroups. Next, we determined the soft-thresholding power for the scale-free network by selecting the power value that yielded the maximum R^2 value. In this case, the power value was determined to be 4. To ensure robustness and reliability, each module was required to contain a minimum of 30 genes. We evaluated the distance between gene pairs using the topological overlap matrix similarity. Hierarchical clustering analysis was performed using both the average method and dynamic method, allowing us to construct a clustering tree and assign genes into specific modules. To gain insight into the functional annotations and pathways associated with the key modules, we conducted Gene Ontology (GO) and Kyoto Encyclopedia of Genes and Genomes (KEGG) analyses. This analysis was performed using the “clusterProfiler” R package, which enabled us to determine the enriched biological processes and pathways associated with the identified key modules. By employing WGCNA and conducting GO and KEGG analyses on the key modules, we can better understand the biological functions and pathways specific to each OA subgroup, providing valuable insights into the molecular underpinnings of these subgroups.

### 2.10 Machine learning screening for hub hedgehog-related genes

To identify important hedgehog-related differentially expressed genes (DEGs), we employed a combination of various algorithms such as lasso, ridge, and elastic net. Additionally, SVM-RFE, random forest, Bagging, GBM, Xgboost-xgbLinear, Xgboost-xgbtree, and Decision Tree methods were utilized to rank the importance of hedgehog-related genes. All machine learning algorithms are set to default parameters. Machine learning code on the public website: https://github.com/gxykdx/OA_hedgehog. Based on the results from these algorithms, we selected the top 20 ranked hedgehog-related genes based on their importance scores. These scores were determined by each algorithm’s respective feature selection or ranking mechanism. To determine hub hedgehog-related genes, we considered the common genes that were selected by at least a certain number of these algorithms. By applying this criterion, we could identify the most consistently important genes across multiple algorithms, providing robust evidence for their significance in the context of the hedgehog pathway. This comprehensive approach allowed us to identify a set of hub hedgehog-related genes that are deemed influential and critical in the regulation and functioning of the hedgehog pathway.

### 2.11 Building predictive models

To construct a predictive model for OA, we performed multiple logistic regression analysis using the hub hedgehog-related genes as predictors. This analysis aimed to identify the relationship between the expression levels of these genes and the presence or absence of OA. To assess the reliability of the constructed model, we utilized the bootstrap method with 1,000 iterations of internal validation. This technique involves resampling the dataset to estimate the model’s performance and evaluate its stability. Furthermore, for external validation of the model, we used the GSE48556 dataset, which provides an independent set of samples to validate the predictive performance of the model on unseen data. To visualize the results, column line plots can be created using the “regplot” R package. These plots enable the visualization of the relationship between the predicted probabilities of OA and the corresponding gene expression levels. Additionally, calibration curves can be generated to evaluate the stability of the model after resampling. These curves provide insights into how well the predicted probabilities align with the observed probabilities, indicating the calibration or accuracy of the model. Lastly, decision curve analysis (DCA) can be employed to assess the clinical utility of the predictive model. DCA helps determine whether the model’s predictions are beneficial in guiding clinical decisions by evaluating the net benefits at different threshold probabilities. By performing these analyses and evaluations, we aim to develop a reliable and clinically relevant predictive model for OA based on the hub hedgehog-related genes.

### 2.12 GSEA of hub hedgehog-related genes

To perform the correlation analysis between the hub hedgehog-related genes, it is necessary to exclude control samples from the analysis. Using the Spearman method, calculate the correlation coefficients between the expression levels of these genes. The Spearman correlation coefficient assesses the monotonic relationship between variables and is suitable for analyzing non-linear associations. Next, obtain the KEGG pathway files from the MSigDB Database database (https://www.gsea-msigdb.org/gsea/msigdb). These pathway files contain comprehensive annotations of biological processes and signaling pathways. Rank the genes based on their correlation coefficients, with higher coefficients indicating stronger associations. This ranking will help prioritize the genes that exhibit the most significant correlations with the hub hedgehog-related genes. Subsequently, perform a gene set enrichment analysis (GSEA) using the “clusterProfiler” R package. The GSEA analysis will evaluate whether the ranked genes are significantly enriched in any specific KEGG pathway. By conducting a significance test, it can be determined if these genes have a statistically significant association with particular biological processes or signaling pathways. This comprehensive analysis combining correlation analysis, gene ranking, and GSEA will provide insights into the potential functional relevance and involvement of the hub hedgehog-related genes in specific molecular pathways and biological processes.

### 2.13 Methylation analysis and genome-wide association study (GWAS analysis)

Download the OA-related DNA methylation dataset, GSE73626, from the GEO database. Then, utilize the “RnBeads” R package to perform genome-wide DNA methylation analysis on the hub hedgehog-related genes. The Gene Atlas database (http://geneatlas.roslin.ed.ac.uk/) is an extensive resource that provides comprehensive documentation on associations between millions of variants and hundreds of traits utilizing the UK Biobank cohort. This database encompasses data from 452,264 individuals in the UK Biobank database, covering a vast array of 778 phenotypes and 30 million loci. We selected “M91-M94 chondropathies” as the traits, followed by the default parameters for all options, and then input genes separately to generate the image.

### 2.14 Single cell analysis

We utilized the Seurat R package to process the single-cell RNA sequencing (scRNA-seq) data. Cells expressing a minimum of 200 genes and a maximum of 2,500 genes were identified. To identify highly variable genes, we employed the “FindVariableGenes” function and conducted principal component analysis (PCA). For single-cell visualization, we employed the uniform manifold approximation and projection (UMAP) method for dimensionality reduction. The resulting single-cell plot was visualized using the “DimPlot” function, while gene expression plots were generated using the “FeaturePlot” function. To annotate cell types, utilize the ‘HumanPrimaryCellAtlasData ()' function from the ‘celldex’ package. Then, calculate the hedgehog scores for each cell type and evaluate the differences in hedgehog scores between the normal and OA groups within the same cell type. Further visualize these differences using the “FeaturePlot” function. The “scMetabolism” R package is used to assess hedgehog activation scores in single cells.

### 2.15 Pseudotime analysis

To perform pseudotime analysis on a subset of chondrocytes, follow these steps:1. Extract a subset of chondrocytes for analysis, selecting 1,357 chondrocytes. 2. Utilize the Monocle R package to perform dimensionality reduction and clustering on the chondrocytes. This package offers specialized functions for single-cell RNA-seq analysis. 3. Prepare the cells for subsequent pseudotime analysis by applying specific criteria. Select cells with mean expressions greater than 0.1 and dispersion empirical values exceeding 1 multiplied by the dispersion fit. These criteria help ensure that the selected cells are suitable for the subsequent analysis. 4. Employ the ‘DDRTree’ method in the reduceDimension function of the Monocle package for dimensionality reduction and ordering. This method helps capture the structure and progression of the cell differentiation trajectory. 5. Visualize the distribution trajectory of the cells using the plot_cell_trajectory function from the Monocle package. This visualization provides insights into the developmental trajectory of the chondrocytes and their differentiation patterns over pseudotime. 6. Analyze the expression changes of the hub hedgehog-related genes among different clusters along the cell differentiation trajectory. This analysis can be performed using Monocle’s built-in tools to assess how the expression of these genes varies as the cells progress through different stages of differentiation. By following these steps, you can gain a deeper understanding of the pseudotime dynamics and expression changes of the hub hedgehog-related genes within the chondrocyte population, providing insights into their role in chondrocyte differentiation and development.

### 2.16 Cellular communication

Extract a subset of chondrocytes and based on the hedgehog scores, define chondrocytes with scores above the 75th percentile as high hedgehog score chondrocytes, and those below the 25th percentile as low hedgehog score chondrocytes. To perform cell communication analysis, utilize the “CellChat”R package. This package allows you to explore and analyze cell-cell communication networks within your dataset.

### 2.17 Real-time PCR analysis

Mouse articular chondrocytes were obtained from Wuhan Procell Life Science and Technology Co., Ltd. The cells were cultured in DMEM/F12 medium supplemented with 10% fetal bovine serum. For the inflammation model, the cells were stimulated with IL-1β at a concentration of 10 ng/mL for 24 h. Total RNA was extracted using the RNAeasy™ Animal RNA Extraction Kit (Beyotime Biotechnology, China), and the quality was assessed through spectrophotometry using the NanoDrop One instrument. Subsequently, reverse transcription was performed to generate cDNA. The extracted RNA was reverse transcribed into cDNA using the PrimeScript™ RT Master Mix (TAKARA, Japan). The cDNA was amplified using the PowerUp™ SYBR^®^ Green Master Mix (Applied Biosystems, USA) on the ABI 7500 Real-Time PCR System. RT-qPCR was conducted to analyze the expression of different genes, with Gapdh serving as an internal reference. The relative mRNA expression levels were calculated using the 2^(-ΔΔCt) method, and statistical significance was determined by a *p*-value less than 0.05. Please refer to Table 1 for the primer sequences.

**TABLE 1:** Primers used in this study.

Primer	Sequence
Gli3-F	GAA​GAA​ACG​CAA​TCA​CTA​TGC​AG
Gli3-R	GTC​CCA​CGG​TAA​GGG​AGA​GA
Csnk1g2-F	AGG​AGT​ACA​TCG​ACC​CTG​AGA
Csnk1g2-R	CTC​TGC​TCT​TTG​CCC​AAG​TG
Wnt5a-F	CAA​CTG​GCA​GGA​CTT​TCT​CAA
Wnt5a-R	CCT​TCT​CCA​ATG​TAC​TGC​ATG​TG
Wnt5b-F	GGA​GTA​CGG​CTA​CCG​CTT​TG
Wnt5b-R	TCC​TCC​GAT​CCC​TTG​GCA​A
Prkx-F	GGG​GCA​CTC​GTT​ACA​AGA​TTG
Prkx-R	CTC​CTT​CAC​CAG​GTT​TAC​ACG
Rab23-F	CCA​CAG​ACA​GGG​AAT​CTT​TTG​AA
Rab23-R	GGT​AGA​ACC​TCA​GCT​TTA​GCC​T
Fbxw11-F	TAC​CAG​AGC​AAG​GCT​TAG​ATC​A
Fbxw11-R	TTC​TTT​CTG​AGA​GTC​CCT​TCC​A
Prkaca-F	GGT​GAC​AGA​CTT​CGG​TTT​TGC
Prkaca-R	CAC​AGC​CTT​GTT​GTA​GCC​TTT
Bmp8b-F	CCG​GGA​CTC​CTA​TGG​CTA​CT
Bmp8b-R	CAT​CCG​TCA​TGG​CAC​GGT​A
Gapdh-F	AAT​GGA​TTT​GGA​CGC​ATT​GGT
Gapdh-R	TTT​GCA​CTG​GTA​CGT​GTT​GAT

## 3 Results

### 3.1 Hedgehog pathway activity rises in OA

**FIGURE 2:**
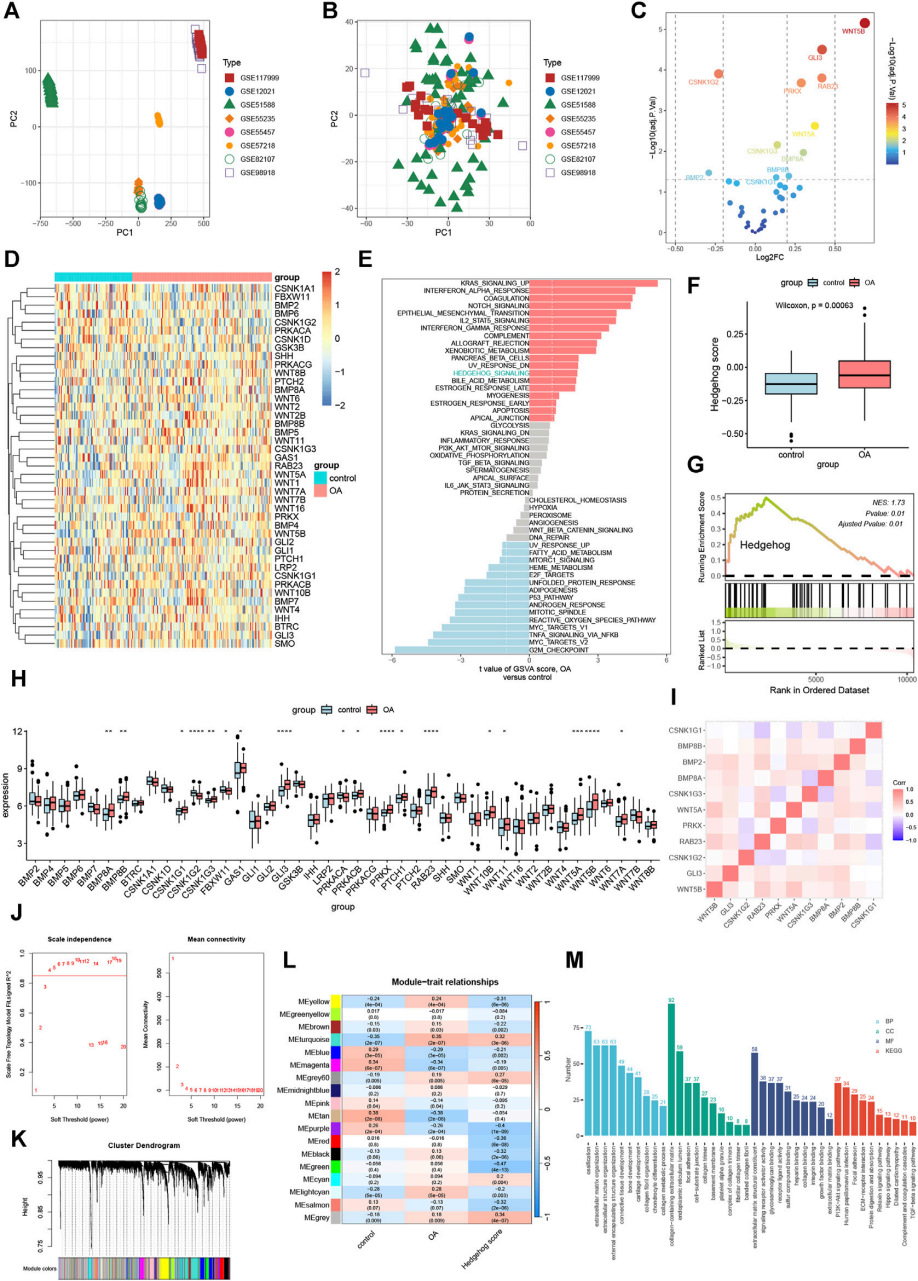
Enrichment Analysis. Principal component analysis of 8 data sets batches before **(A)** and after **(B)** correction. **(C)** Volcano plot showing the results of differential expression analysis of Hedgehog-related genes. **(D)** Heat map of Hedgehog-related genes. **(E)** Gene set variance analysis (GSVA) showing that the Hedgehog pathway is highly enriched in OA. **(F)** SsGSEA shows that the Hedgehog score is significantly upregulated in OA. **(G)** GSEA demonstrates significant activation of Hedgehog in OA (NES: enriched score after standardization). **(H)** Differential analysis of Hedgehog-related genes in OA and control groups. *p < 0.05, **p < 0.01, ***p < 0.001, ****p < 0.0001. **(I)** Correlation analysis of significantly differentially expressed hedgehog-related genes. **(J)** Scale-free fit index analysis of different soft threshold capabilities **(left)** and mean connectivity analysis of various soft threshold capabilities **(right)**. **(K)** Dendrogram of clustering tree of co-expression modules. Different colors correspond to other co-expression modules. **(L)** Heat map showing the correlation of modules with control, OA, and hedgehog scores (each module contains correlation coefficients and corresponding *p*-values). **(M)** Results of GO and KEGG enrichment analysis of turquoise module genes. GO, Gene Ontology; BP, Biological Process; CC, Cellular Component; MF, Molecular Function; KEGG, Kyoto Encyclopedia of Genes and Genomes.

We performed a merged analysis on GSE117999, GSE12021, GSE51588, GSE55235, GSE55457, GSE57218, GSE82107, and GSE98918, resulting in 75 control samples and 135 OA samples, with data available for 10,827 genes. As shown in Figure 2A, the samples from the eight independent datasets exhibited batch effects, but after removing the batch effect, they clustered together (Figure 2B). This indicates that cross-platform normalization successfully eliminated the batch processing effects, enabling further analysis. The results of differential expression analysis showed significant upregulation of WNT5A, WNT5B, GLI3, RAB23, PRKX, CSNK1G3, BMP8A, CSNK1G1B, and G1BMP8B, while CSNK1G2 and BMP2 were significantly downregulated (Figure 2C). The heatmap in Figure 2D illustrates the expression changes of hedgehog-related genes in each sample. Subsequently, we analyzed the activity of the hedgehog pathway in OA. GSVA (Figure 2E), ssGSEA (Figure 2F), and GSEA (Figure 2G) all confirm a significant upregulation of the hedgehog pathway in OA. The results of the rank-sum test (Figure 2H) indicate that BMP8A, BMP8B, CSNK1G1, CSNK1G3, GAS1, GLI3, PRKACB, PRKX, PTCH1, RAB23, WNT11, WNT10B, WNT5A, WNT5B, and WNT7A are significantly overexpressed in OA, while CSNK1G2, FBXW11, and PRKACA show significant downregulation in OA. The correlation heatmap (Figure 2I) demonstrates a positive correlation among most of the hedgehog-related DEGs in OA. Overall, these findings provide valuable insights into the expression changes and activity of the hedgehog pathway in osteoarthritis, highlighting specific genes that are upregulated or downregulated in this disease.

### 3.2 Co-expression network construction

We set the optimal soft threshold as 4 to construct a scale-free network (Figure 2J). Using gene expression data, we computed the correlation between genes within the samples and clustered highly correlated genes into the same module. By conducting correlation analysis between modules and traits, we observed a strong correlation between the turquoise module and the hedgehog score (Figure 2L). Subsequently, we utilized the “clusterProfiler” R package to perform enrichment analysis on the genes within the turquoise module. In terms of Biological Processes (BP), the turquoise module genes exhibited significant enrichment in ossification, extracellular matrix organization, and extracellular structure organization (Figure 2M). Regarding Cellular Components (CC), these genes were significantly associated with collagen-containing extracellular matrix, endoplasmic reticulum lumen, and focal adhesion (Figure 2M). Molecular Function (MF) analysis revealed that the turquoise module genes were markedly enriched in extracellular matrix structural constituents, signaling receptor activator activity, and glycosaminoglycan binding (Figure 2M). Furthermore, KEGG pathway analysis demonstrated significant enrichment of the turquoise module genes in the PI3K-Akt signaling pathway, Human papillomavirus infection, and Focal adhesion (Figure 2M). These findings suggest that the genes within the turquoise module play important roles in various biological processes, cellular components, molecular functions, and signaling pathways related to OA pathogenesis. The enrichment analysis provides insights into the potential mechanisms underlying the involvement of the turquoise module genes in aspects such as ossification, extracellular matrix organization, and PI3K-Akt signaling pathway.

### 3.3 Immuno-infiltration analysis

**FIGURE 3:**
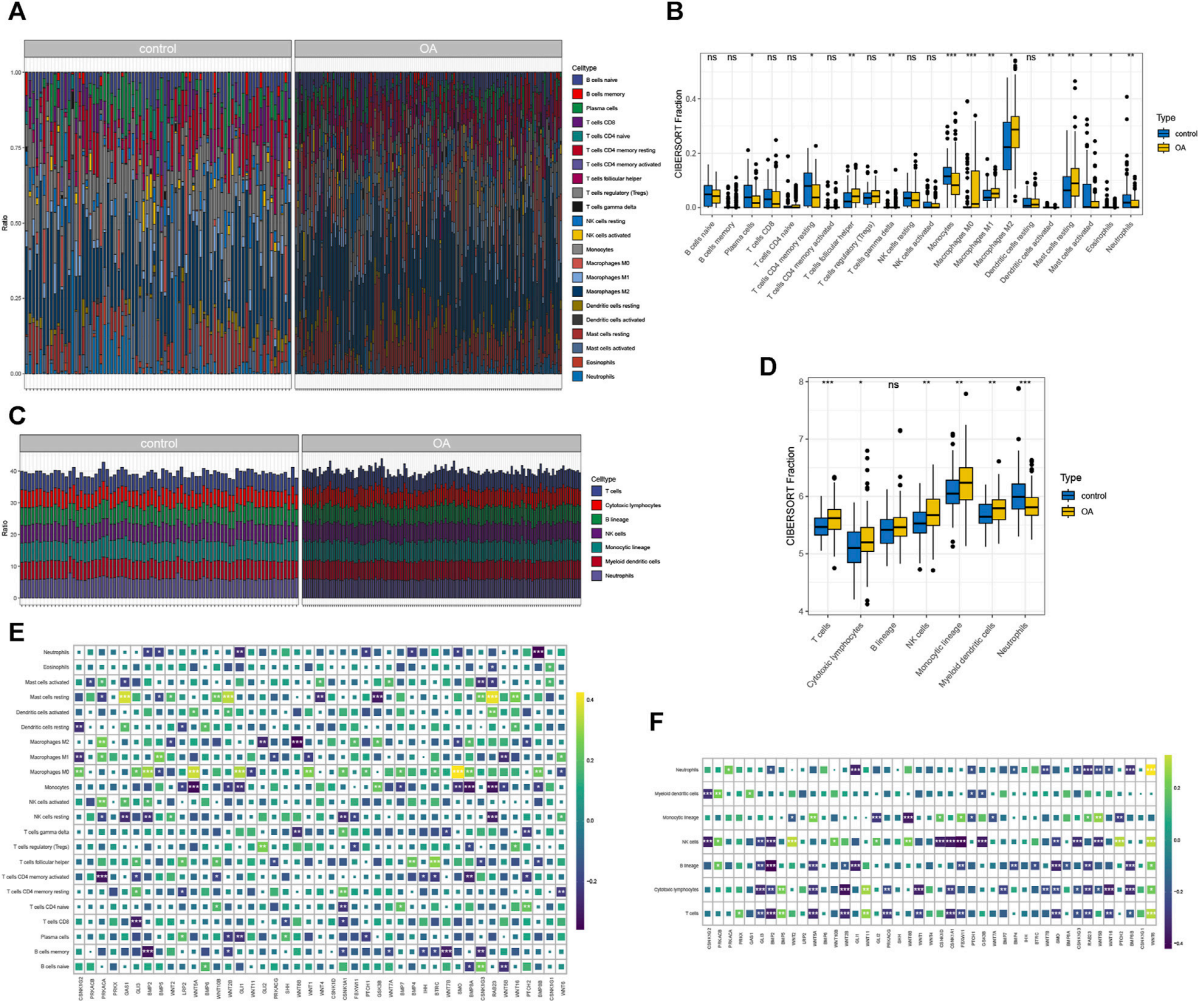
Immune infiltration cibersort **(A)** and the percentage of immune cells calculated by MCP-counter **(B)**; heat map showing the correlation between immune cells and gene expression calculated by cibersort **(C)** and MCP-counter **(D)**; cibersort **(E)** and MCP-counter **(F)** calculated by immune cells in OA group and control for differential expression analysis.

The Cibersort results indicate a significant proportion of M2 macrophages (Figure 3A). The immune infiltration results calculated by MCP-counter are shown in Figure 3B. Correlation analysis reveals significant associations: RAB23 is significantly correlated with 6 types of immune cells, CSNK1G2 is significantly linked to 3 types of immune cells, GLI3 are significantly associated with 5 types of immune cells, and WNT5A and WNT5B are significantly correlated with 3 types of immune cells (Figure 3C). The immune infiltration results from MCP-counter and gene correlations are illustrated in Figure 3D. Differential expression analysis shows significant expression of 13 immune cell types identified by Cibersort (Figure 3E), as well as significant expression of 6 immune cell types identified by MCP-counter (Figure 3F). These findings highlight the presence of specific immune cell types, such as M2 macrophages, and their association with the hub hedgehog-related genes. The results from both Cibersort and MCP-counter provide insights into the immune cell composition and their potential involvement in osteoarthritis. The correlation analysis further emphasizes the significance of immune cell-related genes in the context of OA.

### 3.4 Subtype construction

**FIGURE 4:**
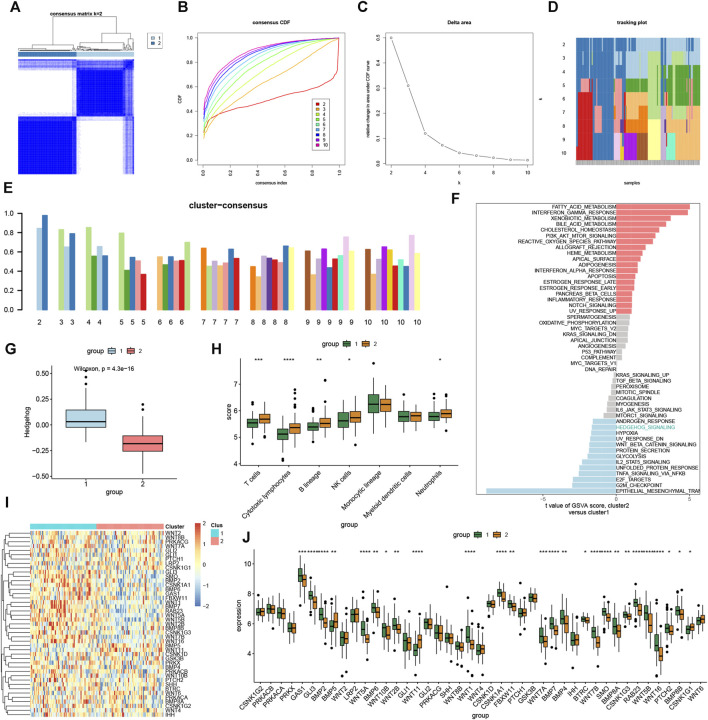
Identification of molecular clusters based on hedgehog-related genes in OA. A Consensus clustering matrix when k = 2 **(A)**. Cumulative distribution function (CDF) curves of clustering **(B)**, CDF delta area curves **(C)**, tracking plot **(D)** and consensus clustering score of each cluster **(E)**, and non-negative matrix heatmap. GSVA **(F)** and ssGSEA **(G)** indicate that cluster1 has higher hedgehog pathway activity than cluster2. **(H)** Immunological infiltration analysis of cluster1 and cluster2. **(I)** Heat map showing hedgehog-related gene expression in each cluster1 and cluster2 sample. **(J)** Differential expression analysis of hedgehog-related genes in cluster1 and cluster2 groups.

To further demonstrate the role of the hedgehog pathway in OA and elucidate the expression patterns related to the immune microenvironment in OA, we applied consensus clustering algorithm to group OA samples based on hedgehog-related genes. Based on the similarity matrix obtained from the consensus matrix, we determined the final subtypes. After considering various factors such as the consensus clustering results, CDF plot, relative changes in CDF curve area, tracking plot, and consistent clustering scores, we identified the optimal value of k = 2. Consequently, we classified the total of 135 patients into two distinct subtypes: subtype 1 with 67 cases and subtype 2 with 68 cases (Figures 4A–E). Considering the heterogeneity of OA patients, significant differences in hedgehog activity between different subgroups can demonstrate variations in the hedgehog pathway among OA patients. GSVA (Figure 4F) and ssGSEA (Figure 4G) algorithms revealed that subtype 2 exhibited low hedgehog activity characteristics. Immune infiltration analysis indicated significant upregulation of T cells, cytotoxic lymphocytes, B lineage, NK cells, and neutrophils in subtype 2 (Figure 4H). The heatmap displays the expression of hedgehog-related genes in subtype 1 and subtype 2 (Figure 4I). Differential analysis (Figure 4J) revealed that GAS1, GLI3, BMP2, WNT5A, BMP6, WNT10B, WNT2B, WNT1, CSNK1A1, FBXW11, WNT7A, BMP7, BMP4, BTRC, WNT7B, SMO, BMP8A, CSNK1G3, RAB23, WNT5B, WNT16, PTCH2, and BMP8B were significantly higher expressed in subtype 1, while BMP5, WNT11, and CSNK1G1 were significantly downregulated in subtype 1. This further validates the higher hedgehog activity in subtype 1. In summary, through consensus clustering analysis, we identified two distinct subtypes of OA based on hedgehog-related gene expression patterns. Subtype 1 demonstrated higher hedgehog pathway activity, while subtype 2 exhibited low hedgehog activity and distinct immune infiltration characteristics. The differential analysis results provided further insights into the specific genes associated with each subtype, highlighting their potential roles in the pathogenesis of OA and supporting the heterogeneity of the disease.

### 3.5 Small molecule drugs

**FIGURE 5:**
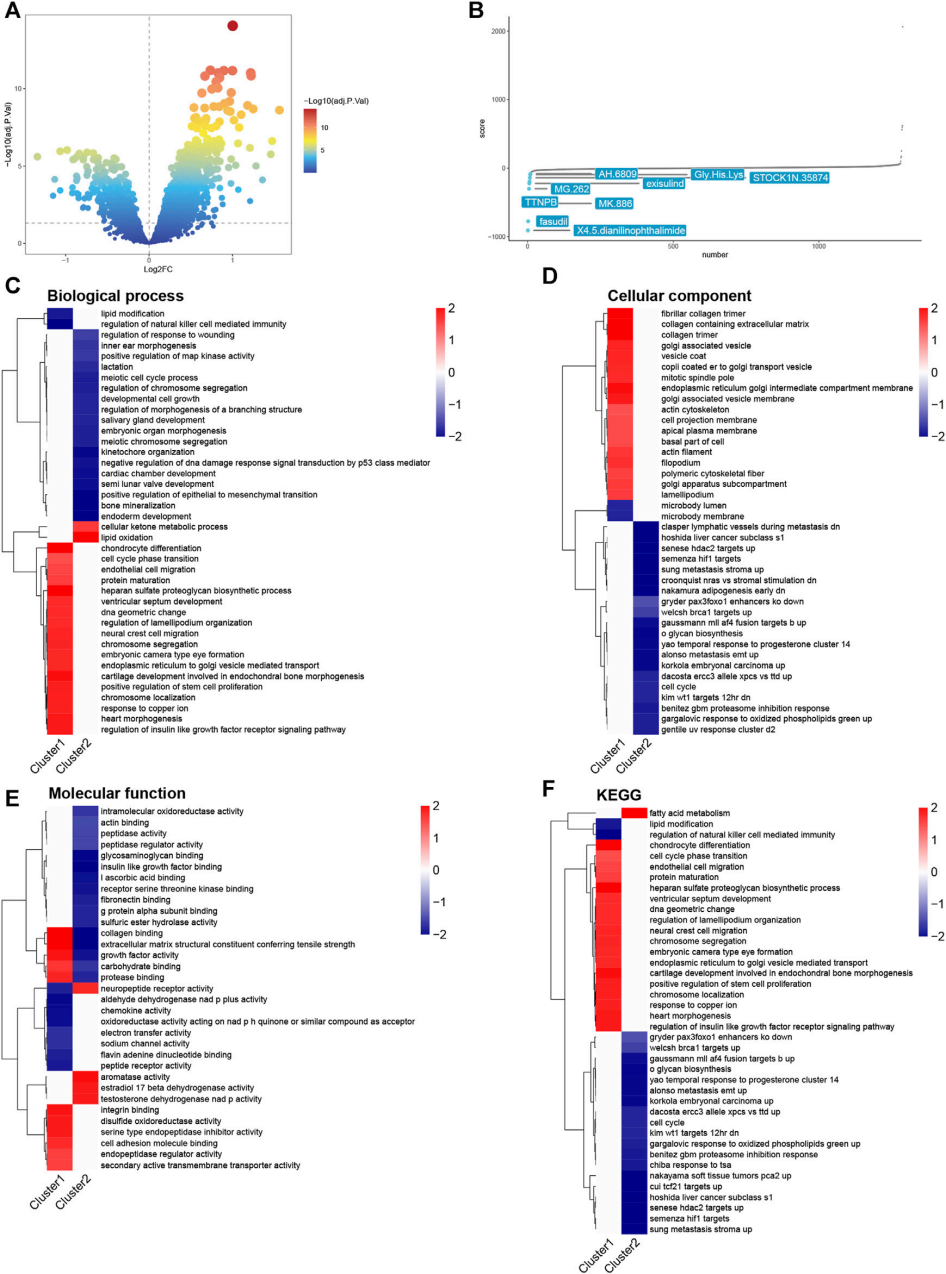
Drug analysis and FGSEA analysis. **(A)** Volcano plot showing the results of differential expression analysis of cluster1 vs cluster2. **(B)** cMap analysis of drugs used to treat subtype1 patients. Lower scores indicate this drug is more likely to inhibit the hedgehog pathway. FGSEA analysis, including BP **(C)**, CC **(D)**, MF **(E)**, and KEGG **(F)**.

The volcano plot indicates that subtype 1 has higher activity compared to subtype 2, with the majority of genes showing significant upregulation (Figure 5A). The cMap drug analysis reveals top drugs for treating patients with high hedgehog activity, including X4.5. dianilinophthalimide, fasudil, TTNPB, MK.886, MG.262, AH.6809, exisulind, Gly. His.Lys, and STOCK1N.35874 (Figure 5B). These findings suggest potential therapeutic options for patients with specific molecular subtypes characterized by their hedgehog pathway activity. Further investigations and validations could be conducted to determine the effectiveness and suitability of these drugs for treating patients within this particular subtype.

### 3.6 FGSEA results

In terms of Biological Processes (BP), chondrocyte differentiation is significantly activated in subtype 1, while lipid oxidation is significantly activated in subtype 2 (Figure 5C). In Cellular Components (CC), subtype 1 is primarily associated with collagen-related processes (Figure 5D). In Molecular Function (MF), collagen binding is significantly activated in subtype 1 and significantly inhibited in subtype 2 (Figure 5E). Regarding the KEGG pathway analysis, subtype 1 is closely related to collagen (Figure 5F). These findings highlight the differences in biological processes, cellular components, molecular functions, and pathways between subtype 1 and subtype 2 of osteoarthritis. Subtype 1 appears to be characterized by chondrocyte differentiation and collagen-related processes, while subtype 2 shows activation of lipid oxidation and distinct molecular functions. The KEGG pathway analysis further supports the association of subtype 1 with collagen-related pathways. These results provide valuable insights into the underlying molecular characteristics and potential mechanisms driving the heterogeneity observed in osteoarthritis subtypes, particularly related to chondrocyte differentiation, collagen metabolism, and lipid oxidation.

### 3.7 Subtype of WGCNA

**FIGURE 6:**
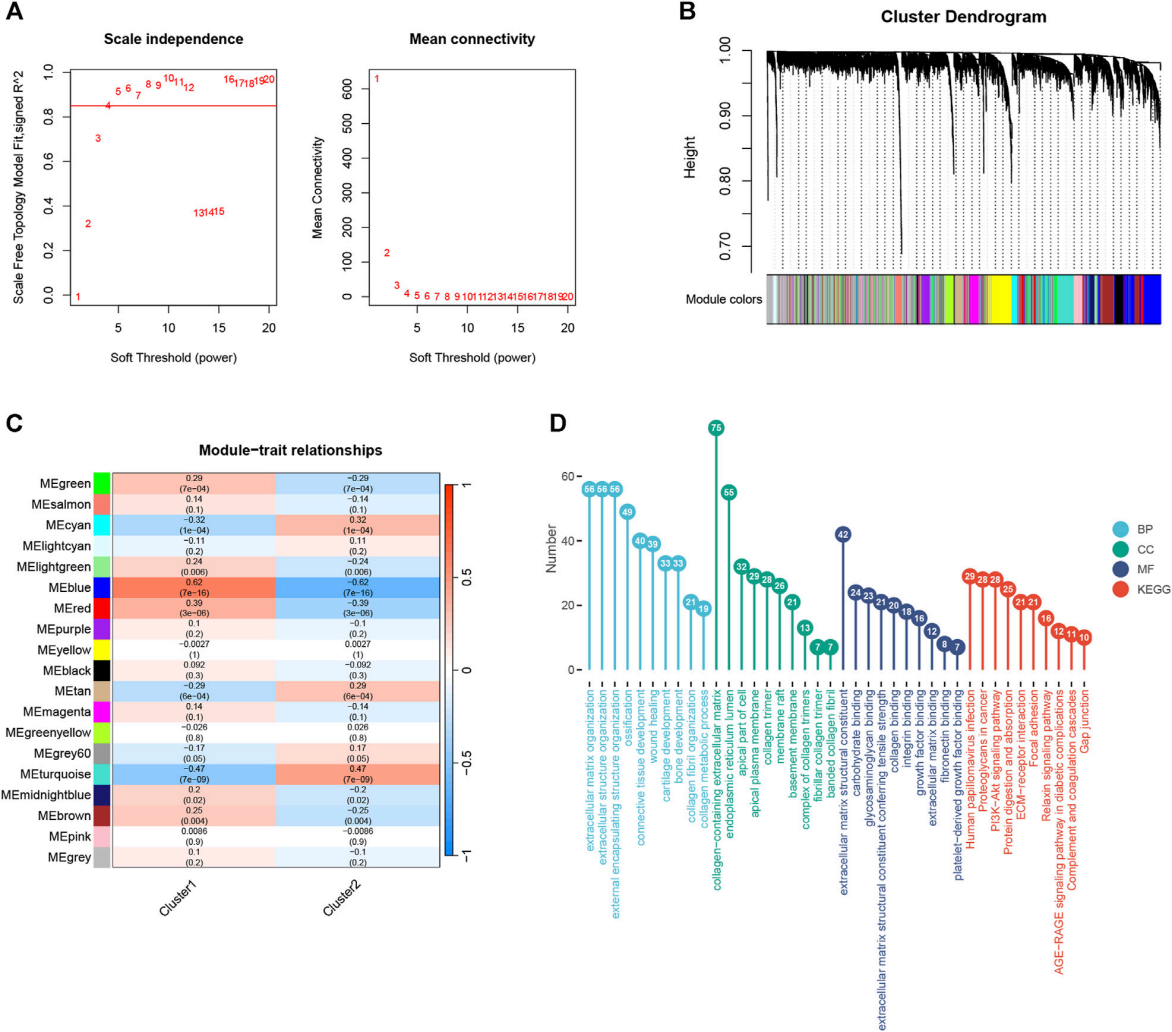
WGCNA analysis of cluster1 and cluster2. **(A)** Scale-free fit index analysis for different soft threshold capabilities **(left)** and average connectivity analysis for various soft threshold capabilities **(right)**. **(B)** Dendrogram of co-expression module clustering tree. Different colors correspond to other co-expression modules. **(C)** Heatmap shows the correlation of modules with cluster1 and cluster2 (each module contains correlation coefficients and corresponding *p*-values). **(D)** Results of GO and KEGG enrichment analyses of blue module genes. GO, Gene Ontology; BP, Biological Process; CC, Cellular Component; MF, Molecular Function; KEGG, Kyoto Encyclopedia of Genes and Genomes.

To identify the key modules most correlated with hedgehog, we performed WGCNA on subtype 1 and subtype 2. Using a correlation coefficient threshold of 0.85, we selected 4 as the soft thresholding power (Figure 6A). Subsequently, a total of 19 modules were generated (Figure 6B). Correlation analysis between modules and traits revealed that the blue module exhibited the most significant correlation with the hedgehog subtype (Figure 6C). We performed enrichment analysis on the genes within the blue module to gain further insights. In terms of BP, the genes in the blue module exhibited enrichment in extracellular matrix organization, extracellular structure organization, and collagen fibril organization (Figure 6D). Regarding CC, these genes were implicated in collagen-containing extracellular matrix, collagen trimer, and complexes of collagen trimers (Figure 6D). MF analysis revealed significant enrichment of the blue module genes in extracellular matrix structural constituents, extracellular matrix structural constituents conferring tensile strength, and collagen binding (Figure 6D). Additionally, KEGG pathway analysis demonstrated associations between the blue module genes and ECM-receptor interaction, PI3K-Akt signaling pathway, and protein digestion and absorption (Figure 6D). The findings from the enrichment analysis of the blue module genes support the involvement of the hedgehog pathway in extracellular matrix and collagen, further substantiating its role in the development and progression of OA. The findings from the enrichment analysis of the blue module genes provide additional evidence for the role of the hedgehog pathway in the development and progression of osteoarthritis, particularly in relation to extracellular matrix organization, collagen metabolism, and relevant signaling pathways such as PI3K-Akt signaling. Overall, these results contribute to our understanding of the biological functions and pathways associated with the key modules most correlated with hedgehog in osteoarthritis subtypes, shedding light on the intricate molecular mechanisms underlying the disease.

### 3.8 Machine learning identification of important hedgehog-related genes and predictive model building

**FIGURE 7:**
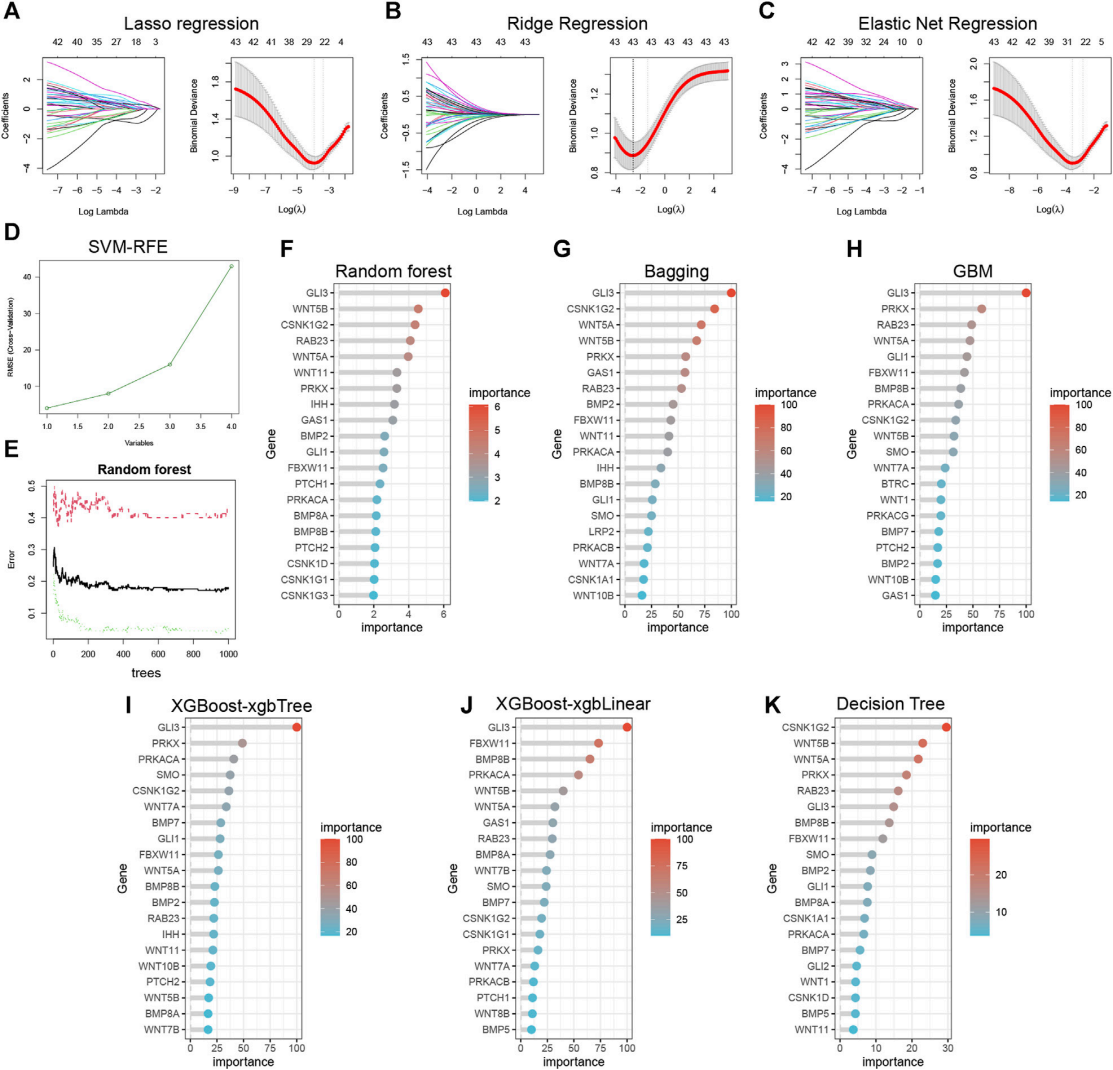
Ten machine learning to identify essential genes. Lasso Regression **(A)**, Ridge Regression **(B)**, and Elastic Net Regression **(C)** identify 26, 43, and 31 variables, respectively, as potential markers for OA. **(D)** Support vector machine-recursive feature elimination (SVM-RFE) process for selecting biomarkers. **(E)** The effect of the decision tree number on the error rate. **(F)** Random forest identification of top 20 essential feature genes. **(G)** Bootstrap aggregating (Bagging), **(H)** Gradient Boosting Machine (GBM), **(I)** eXtreme Gradient Boosting-xgbTree (XGBoost-xgbTree), **(J)** eXtreme Gradient Boosting-xgbLinear (XGBoost-xgbLinear), and **(K)** Decision Tree for top 20 essential feature genes identified respectively.

The lasso (Figure 7A), ridge (Figure 7B), and elastic net (Figure 7C) models successfully identified important hedgehog-related genes. The SVM-RFE results display the accuracy of the model with different numbers of included genes (Figure 7D). By examining the relationship between the number of features and model error, we identified the tree that displayed the lowest error as the final model parameter (Figure 7E). The importance ranking of hedgehog-related genes identified by random forest is shown in Figure 7F. Additionally, using Bagging (Figure 7G), GBM (Figure 7H), Xgboost-xgbLinear (Figure 7I), Xgboost-xgbtree (Figure 7J), and Decision Tree (Figure 7K), we screened out the top 20 hedgehog-related genes based on their importance ranking. These analyses and rankings help prioritize the most influential genes related to the hedgehog pathway, providing valuable information for further investigations and potential therapeutic targeting.

### 3.9 Predictive model construction

**FIGURE 8:**
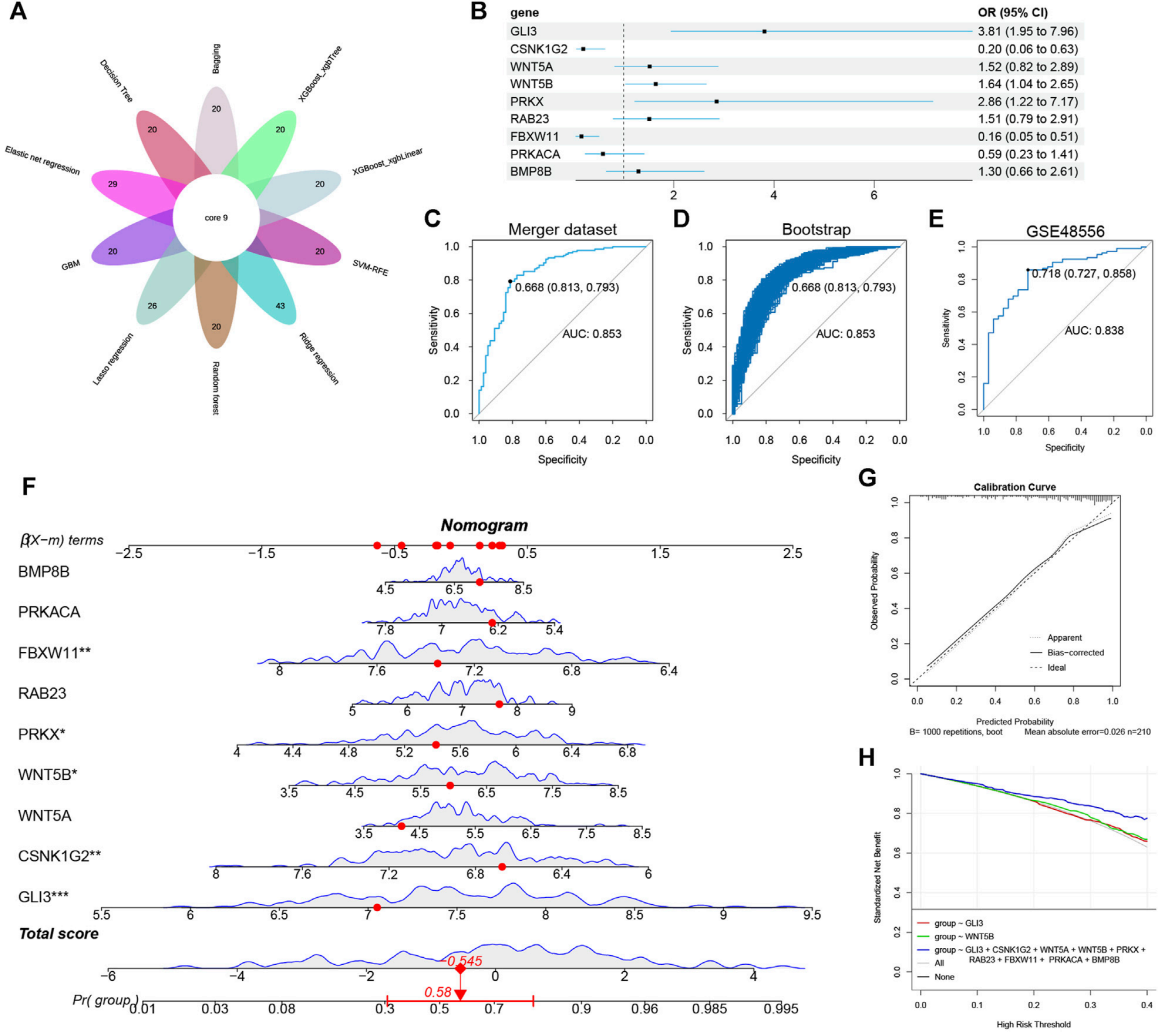
Construction of nomogram model. **(A)** Ten machine learning identified nine hub hedgehog-related genes. **(B)** Forest plot showing the odds ratio and 95% confidence intervals for predicting OA of each gene. **(C)** ROC curves for predicting OA at merge dataset. **(D)** Internal validation of model reliability at merge dataset using Bootstrap. **(E)** GSE48556 externally validates the reliability of the model. **(F)** Nomogram for joint diagnosis of OA based on BMP8B, PRKACA, FBXW11, RAB23, PRKX, WNT5A, WNT5B, CSNK1G2 and GLI3. **(G)** Calibration curve for nomogram validation. **(H)** Decision curve analysis based on the nomogram model.

By analyzing the intersection of 10 algorithms (Figure 8A), we obtained 9 hub hedgehog-related genes (CSNK1G2, GLI3, WNT5A, WNT5B, PRKX, RAB23, FBXW11, PRKACA, and BMP8B). The forest plot displays the results of multivariable logistic regression for these 9 hub hedgehog-related genes (Figure 8B). Next, we assessed the predictive ability of the model constructed using these 7 important hedgehog-related genes. The results showed an AUC value of 0.853 (Figure 8C). Internal validation using the bootstrap method demonstrated the reliability of the model (Figure 8D). External validation using GSE48556 yielded an AUC of 0.838 (Figure 8E), confirming the model’s good stability. The nomogram assigns a score to each value of the feature variable, and the total score is calculated by summing up the scores from all feature variables. This cumulative score reflects the risk of developing osteoarthritis (OA) (Figure 8F). The calibration curve serves to validate the accuracy of the nomogram in diagnosing OA (Figure 8G). Furthermore, the decision curve analysis (DCA) demonstrates that the clinical application of this nomogram offers specific clinical benefits for patients with OA (Figure 8H). Collectively, these findings indicate a close association between these hub hedgehog-related genes and OA. Overall, these findings indicate a close association between the identified hub hedgehog-related genes and osteoarthritis. The constructed model shows good predictive ability, reliability, stability, and potential clinical benefits, further supporting the importance of the hedgehog pathway in the pathogenesis of OA.

### 3.10 Single gene GSEA results

**FIGURE 9:**
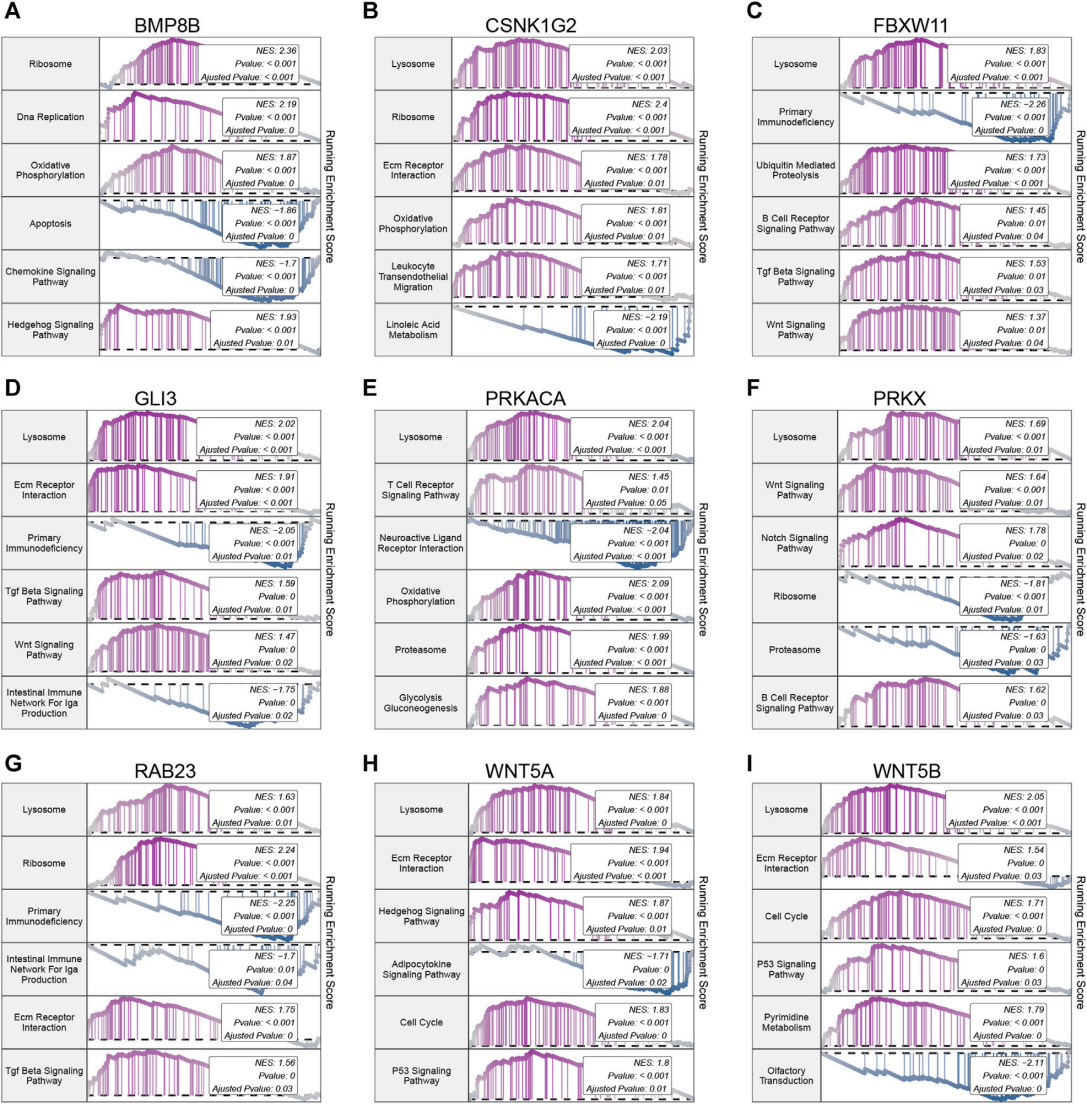
Gene set enrichment analysis (GSEA) of 9 hub hedgehog-related genes. GSEA analysis of BMP8B **(A)**, CSNK1G2 **(B)**, FBXW11 **(C)**, GLI3 **(D)**, PRKACA **(E)**, PRKX **(F)**, RAB23 **(G)**, WNT5A **(H)**, and WNT5B **(I)** in KEGG.

Further exploration of the potential mechanisms of the 9 hub hedgehog-related genes in OA revealed interesting findings. KEGG pathway analysis indicates that 8 out of the 9 hub hedgehog-related genes are associated with lysosomes, including BMP8B (Figure 9A), CSNK1G2 (Figure 9B), FBXW11 (Figure 9C), GLI3 (Figure 9D), PRKACA (Figure 9E), PRKX (Figure 9F), RAB23 (Figure 9G), WNT5A (Figure 9H), and WNT5B (Figure 9I). Only BMP8B is not associated with lysosomes. The association of these hub hedgehog-related genes with lysosomes highlights the potential involvement of lysosomal pathways in OA pathogenesis. These findings suggest that the identified hub hedgehog-related genes may contribute to the development and progression of OA through their involvement in lysosomal processes. Further investigations into the specific roles of these genes in lysosomal pathways could provide valuable insights into the underlying mechanisms of OA and potentially offer new therapeutic targets for the disease.

### 3.11 Methylation and GWAS analysis

**FIGURE 10:**
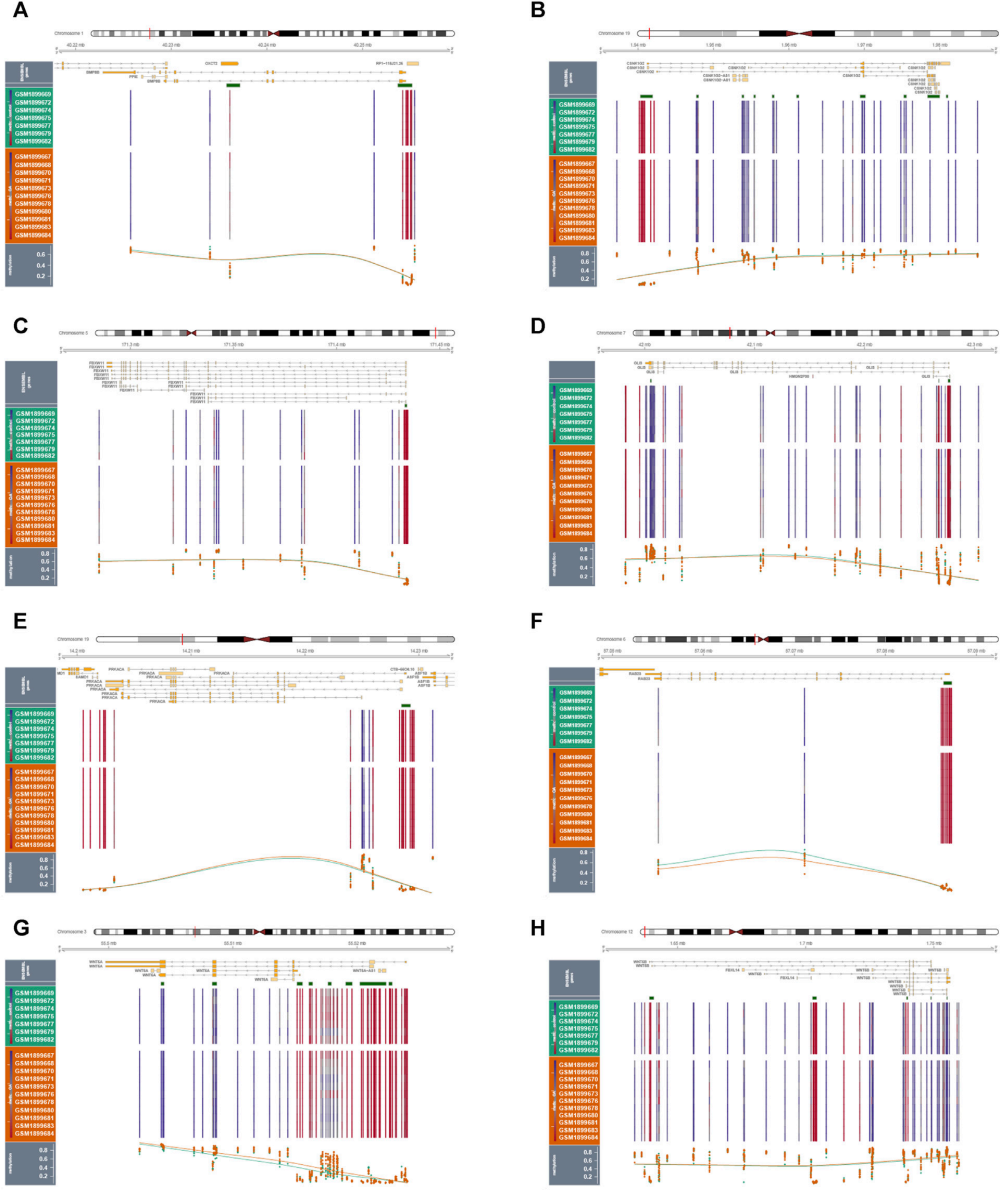
Results of methylation analysis of 9 genes in normal and OA samples **(A-I)**.

By performing methylation analysis using the GSE73626 dataset, we discovered the methylation patterns of 8 hub hedgehog-related genes, including BMP8A (Figure 10A), CSNK1G2 (Figure 10B), FBXW11 (Figure 10C), GLI3 (Figure 10D), PRKACA (Figure 10E), RAB23 (Figure 10F), WNT5A (Figure 10G), and WNT5B (Figure 10H). By analyzing their chromosomal loci information, we further revealed their genetic information (Supplementary Figure S1A). Moreover, by analyzing GWAS data, we identified the single nucleotide polymorphism (SNP) disease regions associated with the 8 hub hedgehog-related genes (Supplementary Figure S1B-I). These findings contribute to our understanding of the epigenetic regulation and genetic information of the hub hedgehog-related genes in the context of osteoarthritis. The methylation patterns shed light on potential regulatory mechanisms, while the analysis of chromosomal loci and SNP disease regions provide valuable genetic information associated with these genes. Overall, these analyses enhance our knowledge of the molecular characteristics and potential genetic contributions of the hub hedgehog-related genes in osteoarthritis, facilitating further investigations into their functional roles and potential implications in disease pathogenesis.

### 3.12 Single-cell quality control and dimension reduction clustering

**FIGURE 11:**
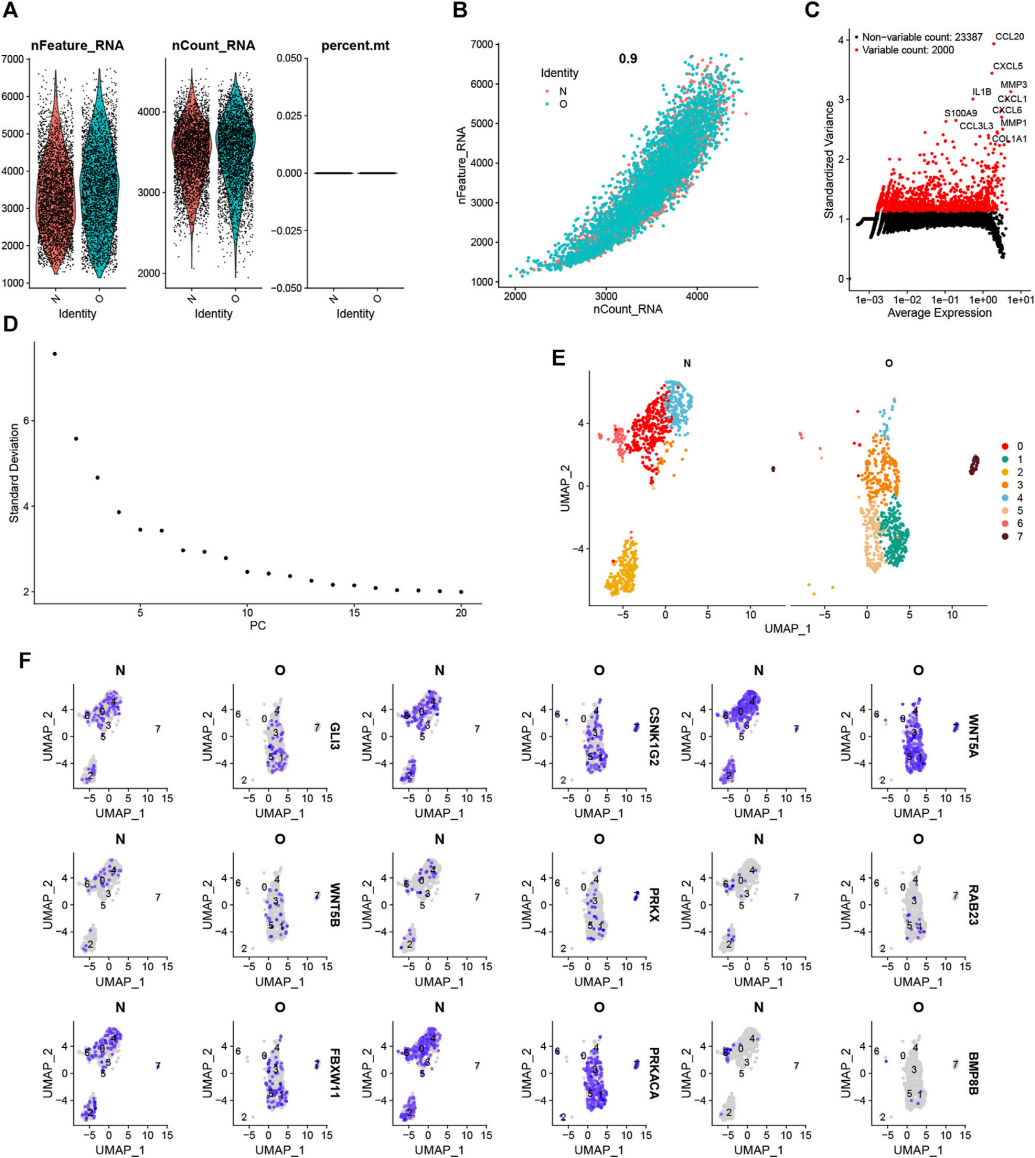
Single-cell clustering for quality control and dimensionality reduction. **(A)** Number of genes, number of molecules, and proportion of mitochondrial genes in each sample. n_Feature indicates the number of genes in each cell. n_Count indicates the number of molecules in each cell. **(B)** Correlation coefficients between the number of genes and the number of molecules. **(C)** The 3,000 highly variable genes are indicated in red, and the ten most essential genes are highlighted. **(D)** Elbow graph of PCA. **(E)** Degradation and clustering analysis. **(F)** The UMAP plots of 9 hub genes in each cluster.

Quality control procedures were performed on the single-cell dataset. The expression profiles for each sample are shown in Figure 11A. Correlation analysis shows high reliability of data (Figure 11B). Subsequently, we identified 2000 highly variable genes and highlighted the top 10 most important genes (Figure 11C). All highly variable genes are highlighted in red in Figure 11C. After dimensionality reduction, 9 principal components (PCs) (Figure 11D) were imported into UMAP for visualization (Figure 11E). The UMAP plot displays the expression of 9 important hedgehog-related genes in various clusters (Figure 11F). Overall, these quality control procedures and visualizations provide a comprehensive understanding of the single-cell dataset, ensuring the reliability of the data and offering valuable insights into the expression patterns and heterogeneity of hedgehog-related genes in different cell populations or clusters.

### 3.13 Cell type annotation

**FIGURE 12:**
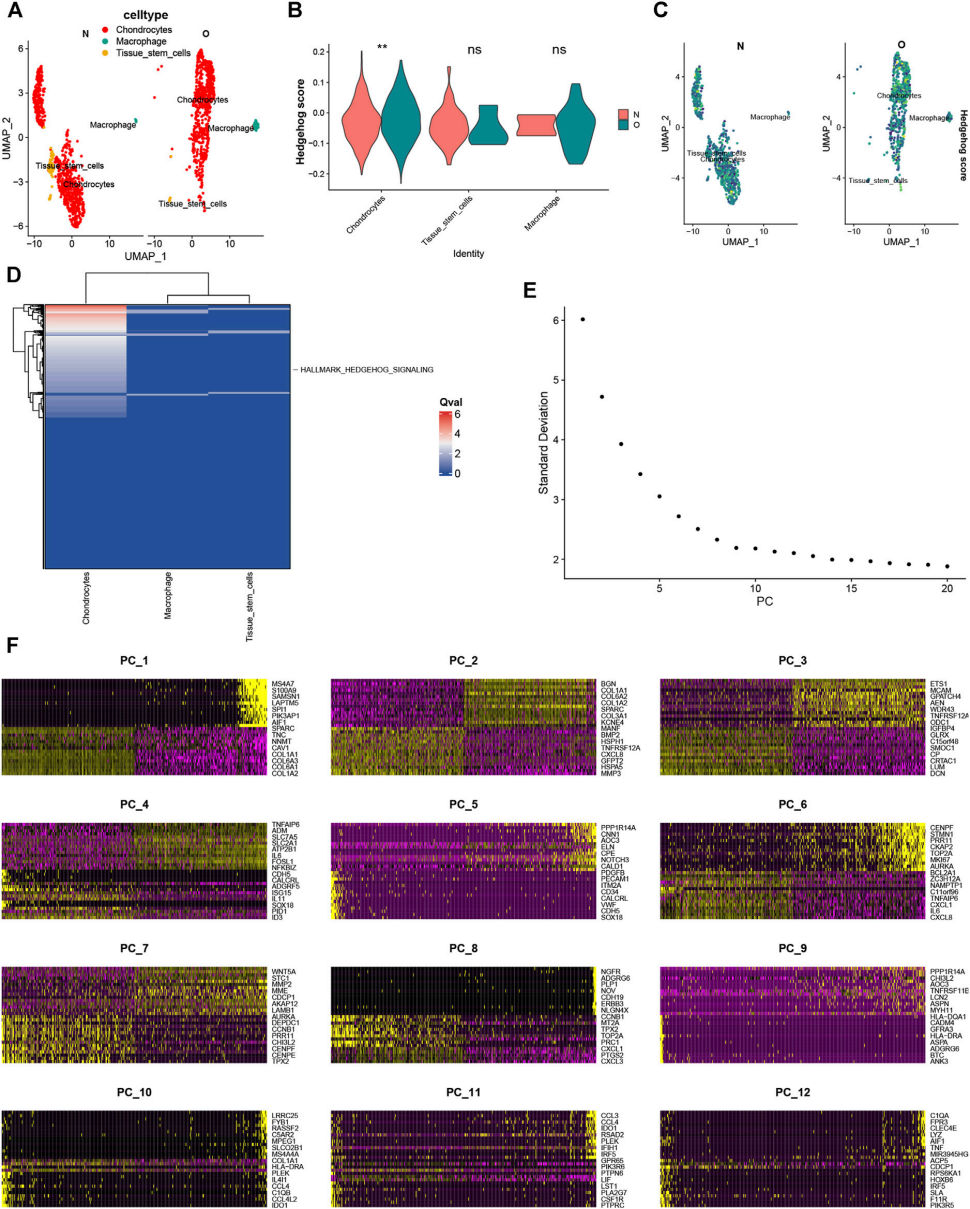
**(A)** Cell annotation revealed chondrocytes, macrophages, and tissue stem cells. **(B)** Hedgehog scores were found to be significantly upregulated in chondrocytes in the OA and normal groups. **(C)** UMAP plot of Hedgehog scores. **(D)** Single-cell pathway analysis (SCPA) found that the Hedgehog pathway was elevated in the OA group. **(D)** Re-dimensionalization analysis after extraction of chondrocytes. **(E)** Nine PCs were imported into UMAP **(F)** The significant genes across the 12 clusters.

Using SingleR for annotation, we identified 3 cell subtypes, including chondrocytes, macrophages, and tissue stem cells (Figure 12A). The expression percentage of hedgehog scores in each cluster is displayed in violin plots (Figure 12B) and UMAP plots (Figure 12C), showing significantly elevated expression of hedgehog scores in OA chondrocytes. hedgehog activation fraction was significantly upregulated in OA (Figure 12D). Therefore, we extracted chondrocytes for further dimensionality reduction. Nine PCs were imported into UMAP for visualization (Figure 12E). Figure 12F displays the significant genes across the 12 clusters.

### 3.14 Pseudotime analysis

**FIGURE 13:**
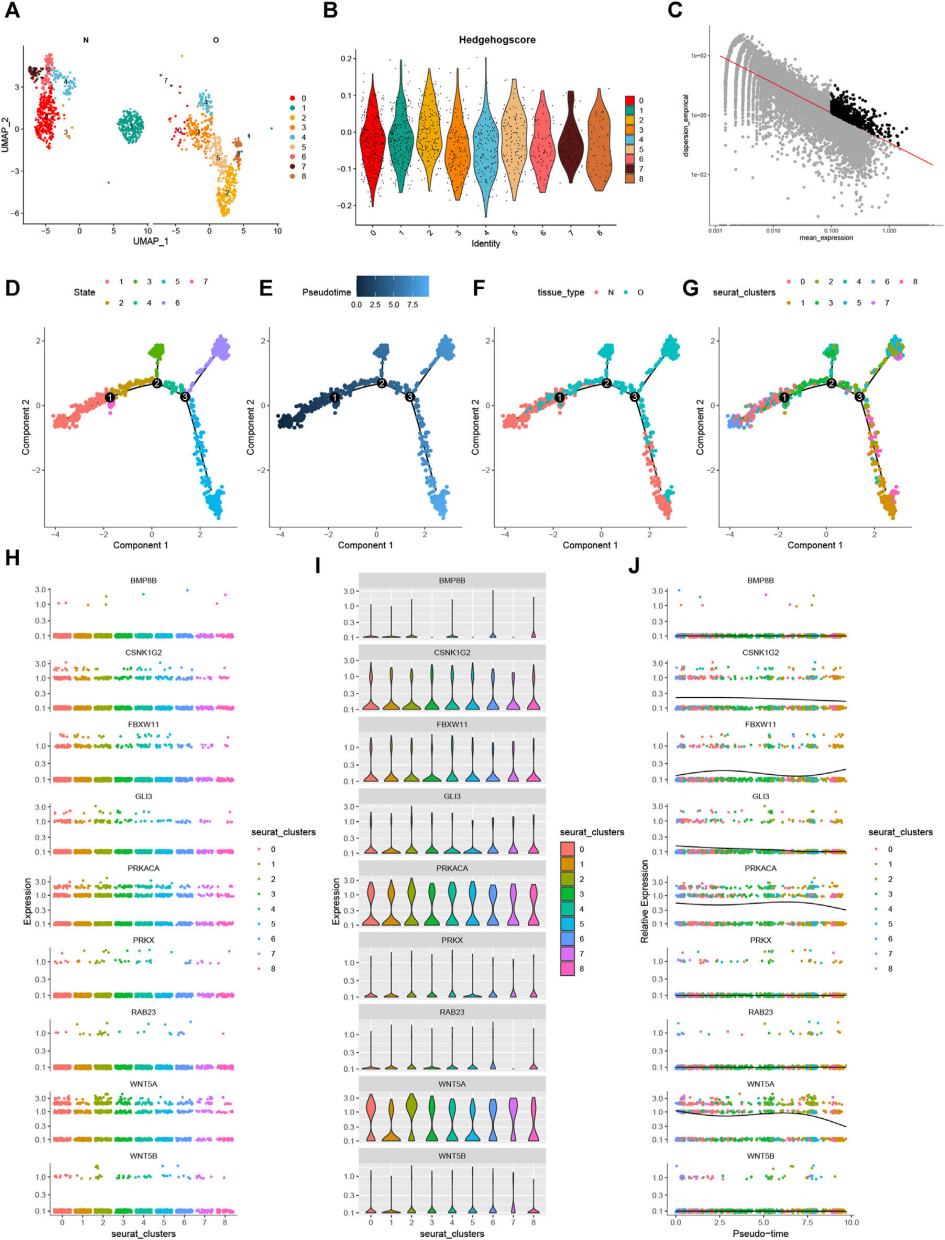
Pseudotime Analysis. **(A)** UMAP of chondrocytes after re-dimensionalization and clustering. **(B)** Hedgehog fraction of each chondrocyte subpopulation. **(C)** Highly variable genes were selected for analysis. **(D)** Seven states of chondrocyte differentiation. **(E)** Differences in the time series of cell differentiation. Dark blue indicates earlier differentiation. Light blue shows later differentiation. **(F)** Differences in chondrocyte differentiation in OA and normal groups. **(G)** All chondrocytes were differentiated into 8 clusters. **(H–J)** Different visualizations show the changes of 9 genes in the pseudotime process.

By extracting the chondrocyte subset for further dimensionality reduction, we discovered 9 chondrocyte subtypes (Figure 13A). Figure 13B displays the Hedgehog scores for each chondrocyte subtype. Using the “FindMarkers” method, we identified significantly differentially expressed genes between OA and normal cells (Figure 13C). Furthermore, we simulated the differentiation trajectory of all chondrocytes and visualized it in Figure 13D, where chondrocytes were labeled with different colors representing seven distinct differentiation states. Our observations revealed a correlation between the intensity of the blue color and the timing of cell differentiation. Specifically, chondrocytes exhibited a left-to-right differentiation pattern over time, with the lightest shade of blue indicating the most recently differentiated cells (Figure 13E). Furthermore, our examination of the differentiation process in both OA and normal chondrocytes unveiled a delay in the differentiation of OA chondrocytes compared to their normal counterparts (Figure 13F). All analyzed cells were confirmed to be chondrocytes (Figure 13G). During the process of cell differentiation, the expression of CSNK1G2, FBXW11, GLI3, PRKACA, and WNT5A changed, while BMP8B, RAB23, WNT5B, and PRKX showed no significant changes in expression (Figures 13H–J). These findings provide insights into the heterogeneity of chondrocytes, the differential gene expression between OA and normal chondrocytes, and the dynamics of chondrocyte differentiation. They highlight potential disruptions in the differentiation process in OA chondrocytes and contribute to our understanding of the molecular mechanisms underlying osteoarthritis at the single-cell level.

### 3.15 CellChat

**FIGURE 14:**
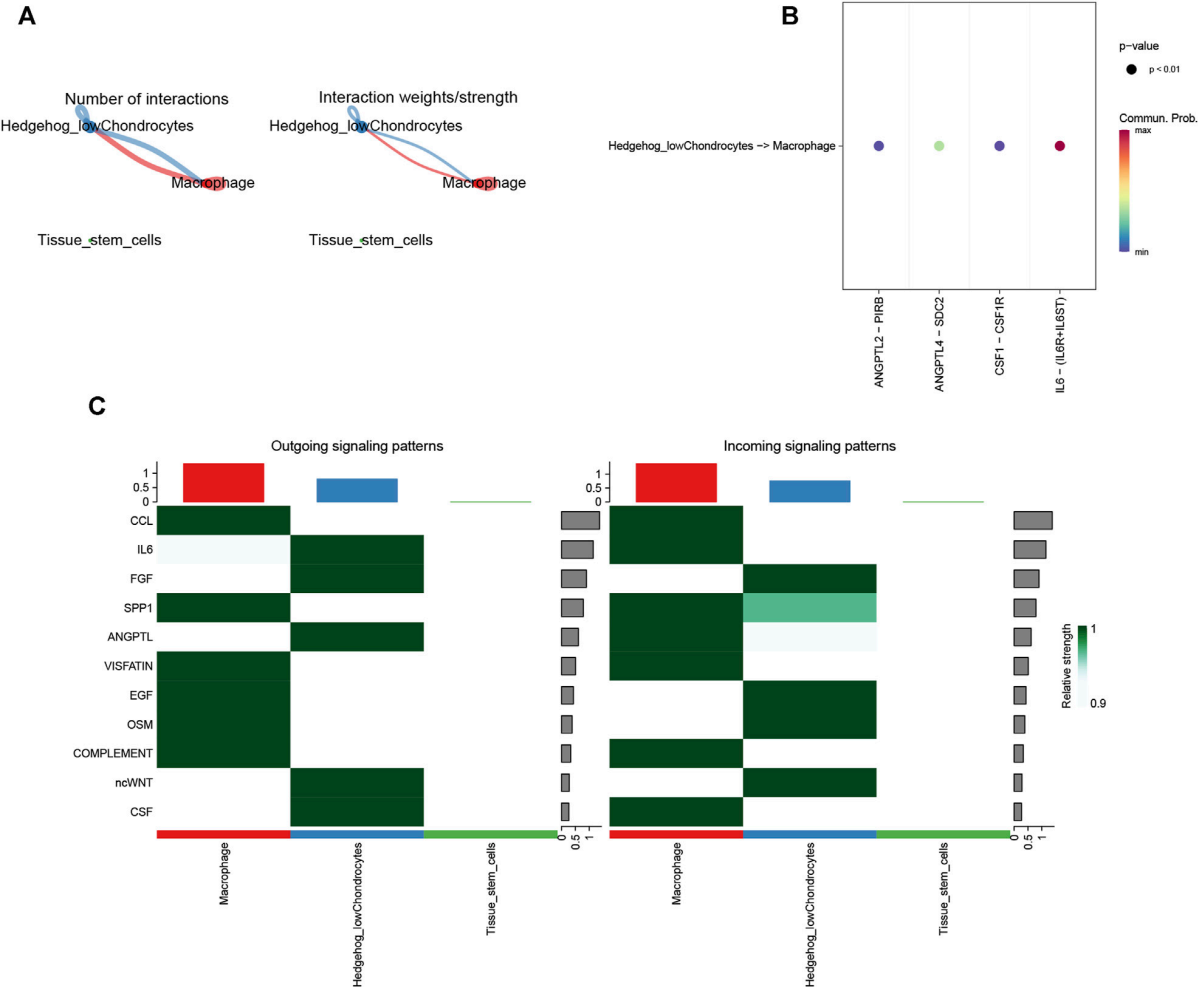
CellChat analysis. **(A)** Statistical analysis of the number and intensity of cellular interactions. **(B)** Ligand-receptor-mediated cellular interactions. **(C)** Cellular signal flow patterns. The horizontal axis is the cell type, and the vertical axis is the pathway. The left panel shows the lightness of the signals sent by each path in each cell type, and the right panel shows the strength of the signals received by each pathway in each cell type.

By cell communication analysis, we found that low hedegehog score chondrocytes and other cell types are associated (Figure 14A). We also analyzed the receptor ligands and signaling pathways that mediate the association of low hedegehog score chondrocytes and macrophages (Figure 14B). Finally, we identified the 11 signals that contribute most to the output and input signals of the cellular taxa (Figure 14C). Overall, these analyses shed light on the communication and interaction networks between different cell types, particularly focusing on low hedgehog score chondrocytes and their associations with other cell populations. The exploration of receptor-ligand pairs, signaling pathways, and key signals contributes to our understanding of the cellular communication landscape and its implications in the context of osteoarthritis.

### 3.16 The result of real-time PCR

**FIGURE 15:**
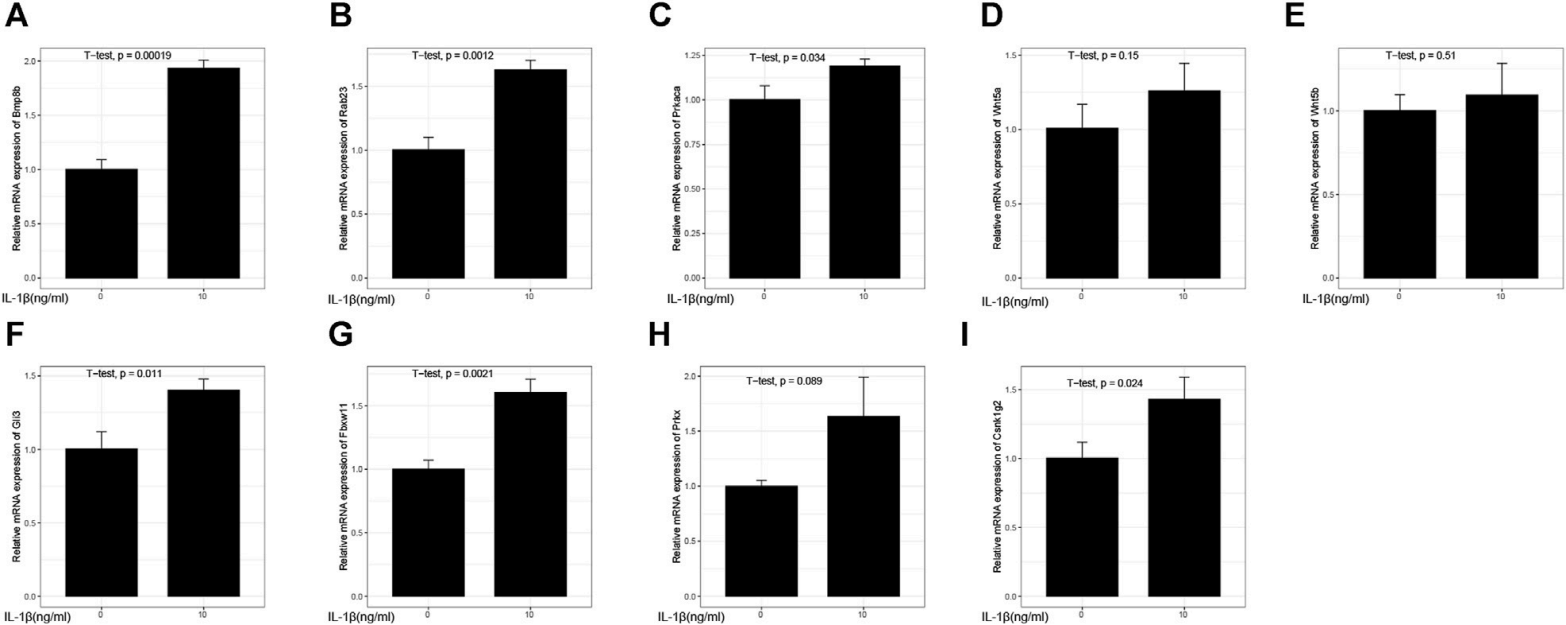
Relative mRNA expression. **(A)** Relative mRNA expression of Bmp8b; **(B)** Relative mRNA expression of Rab23; **(C)** Relative mRNA expression of Prkaca; **(D)** Relative mRNA expression of Wnt5a; **(E)** Relative mRNA expression of Wnt5b; **(F)** Relative mRNA expression of Gli3; **(G)** Relative mRNA expression of Fbxw11; **(H)** Relative mRNA expression of Prkx; **(I)** Relative mRNA expression of Csnk1g2.

Except for the experimental results of Wnt5a (Figure 15D), Wnt5b (Figure 15E), and Prkx (Figure 15H) which were not statistically significant, the inflammation model groups of Bmp8b (Figure 15A), Rab23 (Figure 15B), Prkaca (Figure 15C), Gli3 (Figure 15F), Fbxw11 (Figure 15G), Prkx (Figure 15H), and Csnk1g2 (Figure 15I) gene expression levels were higher than the group without IL-1β treatment.

## 4 Discussion

OA is a multifactorial disease that can lead to chronic joint failure (Latourte et al., 2020). One of its hallmarks is the progressive degradation of the extracellular matrix in chondrocytes. Additionally, calcification and hypertrophic differentiation of chondrocytes within articular cartilage are also characteristic features of OA (1). Currently, the clinical management of OA primarily involves palliative measures such as pain relief and anti-inflammatory medications. However, there is still no approved drug by regulatory authorities that significantly improves OA (Grandi and Bhutani, 2020). Hedgehog signaling is essential for development, crucial for normal anatomical arrangement and activated during tissue damage repair. Dysregulation of hedgehog signaling is associated with OA (Smith et al., 2023). The Hedgehog ligand binds to Patched homolog 1 (PTC), a conserved receptor that activates the GLI transcription factor family. PTC is involved in development, diseases, and bone repair processes. During embryonic development, Hedgehog signaling contributes to limb patterning and plays a crucial role in regulating chondrocyte differentiation and osteogenesis during longitudinal growth of long bones. In skeletal repair and regeneration processes, this signaling pathway also regulates mesenchymal cell differentiation. In the pathogenesis and degenerative process of OA, the Hedgehog signaling pathway is dysregulated (Alman, 2015). Studies have reported increased activation of the Hh pathway in human and murine knee joint cartilage, and the level of this activated pathway is associated with the phenotype of articular cartilage (Bao et al., 2017). This study, based on Bulk RNA-seq and scRNA-seq, systematically explores the mechanisms of the hedgehog pathway in OA. It successfully develops a predictive model and molecular subtypes based on hedgehog-related genes for OA.

In our current study, we found that the hedgehog pathway is significantly activated in OA through ssGSEA, GSVA, and GSEA algorithms. To gain a comprehensive understanding of hedgehog-related gene expression in normal and OA samples, we conducted a systematic analysis. Our analysis revealed differential expression of 18 hedgehog-related genes in normal samples, suggesting the potential involvement of the hedgehog pathway in OA pathogenesis. To delve deeper into the mechanisms underlying the impact of the hedgehog pathway on OA development, we employed WGCNA. Through this approach, we identified the turquoise module, which displayed a significant positive correlation with both the hedgehog score and OA. Enrichment analysis of the turquoise module further highlighted a strong association between the hedgehog pathway and the ECM. The ECM is composed of various macromolecules, traditionally classified as collagen, elastin, fibrillin, proteoglycans (including hyaluronic acid), and non-collagenous glycoproteins (Järveläinen et al., 2009). The abundant ECM forms the avascular and aversive tissue of articular cartilage (Jeon et al., 2018). Under physiological conditions, chondrocytes display minimal mitotic activity and maintain low turnover of collagen. This function is achieved through the ECM’s ability to protect chondrocytes from interacting with components in the pericellular matrix (Glyn-Jones et al., 2015). Hypertrophic chondrocytes can disrupt the ECM, exacerbating the condition of patients with OA (Shi et al., 2019). In OA, Typically, hedgehog activation results in the upregulation of hypertrophic markers, such as type X collagen, along with increased production of nitric oxide and prostaglandin E2. Furthermore, it contributes to the synthesis of various matrix-degrading enzymes, including matrix metalloproteinases and disintegrin with thrombospondin motifs containing proteins. These molecular changes ultimately lead to cartilage degeneration and promote the development of OA (12). Primary cilia are essential for mechanobiological signaling in chondrocytes, and their interaction with the extracellular matrix is crucial for maintaining cartilage homeostasis. Dicam, as a regulator of primary cilia-mediated Indian hedgehog (Ihh) signaling in chondrocytes, can promote proliferation and maturation of growth plate chondrocytes both *in vivo* and *in vitro* (Han et al., 2018). The regulation of Hedgehog signaling in mechanically sensitive bone marrow mesenchymal stromal cells offers a new approach to modeling skeletal diseases and provides new opportunities for targeted therapies against the extracellular matrix (Ghuloum et al., 2022). Given the close relationship between the ECM and OA, it is possible that the hedgehog signaling pathway affects OA development by influencing ECM involvement.

Furthermore, to further validate the association between hedgehog-related genes and OA, we identified 9 hub hedgehog-related genes using 10 machine learning algorithms. The predictive model constructed based on these 9 hub hedgehog-related genes exhibited good performance in predicting OA. Several of these genes have previously been associated with OA. Wnt5a and Wnt5b play a role in coordinating chondrocyte proliferation and differentiation by influencing the expression of cyclin D1, p130, and the chondrocyte-specific Col2a1 to varying degrees. This indicates that Wnt5a and Wnt5b regulate the transition between different regions of chondrocytes, controlling their progression (Yang et al., 2003). During the onset of chondrocyte hypertrophy, Wnt5a serves as a transcriptional target of Gli3 and Trps1 (Wuelling et al., 2020). Notably, the expression of Wnt5a in articular cartilage has been found to be positively correlated with the progressive injury observed in knee joint OA (Li et al., 2016). Increased expression of genes involved in endochondral ossification, such as BMP8B, is characteristic of osteophyte chondrocytes (Gelse et al., 2012). Through GSEA analysis, we found that all 9 genes are associated with lysosomes. Lysosomes are essential cellular organelles involved in the degradation, turnover, and programmed cell death of macromolecules and organelles. Studies have shown that lysosomal dysfunction leads to chondrocyte apoptosis through BAX-mediated mitochondrial damage and cytochrome release (Ansari et al., 2021). The mechanisms of action for other genes in OA are yet to be discovered, indicating the novelty of our findings and suggesting that further experimental exploration of their mechanisms is warranted in the future.

In recent years, increasing evidence has highlighted the crucial involvement of the immune system in both the development and progression of OA. Abnormal loading within joint tissues or systemic factors originating from adipose tissue can trigger the innate immune system, resulting in two forms of low-grade inflammation termed mechanical inflammation and interstitial inflammation. This chronic inflammatory state weakens the joint tissues and raises their vulnerability to damage induced by loading, thereby initiating OA (Berenbaum et al., 2018). To assess the infiltration of immune cells in samples from both OA and normal conditions, we employed CIBERSORT and MCP-counter methodologies. We found that multiple immune cells are closely associated with important biological processes in OA. For example, macrophages were significantly increased in OA, consistent with previous research. As macrophage infiltration increases, the degree of synovial angiogenesis in OA also increases (Haywood et al., 2003). After joint injury, synovial macrophages promote osteophyte formation and other OA-related pathologies such as fibrosis by producing growth factors including bone morphogenetic protein and transforming growth factor-β. These findings highlight the close relationship between immune cells and OA and provide insights into the crucial biological processes involved. Understanding the immune mechanisms underlying OA can potentially lead to the development of targeted therapeutic strategies for this debilitating condition (Jeon et al., 2018).

The advancement of precision medicine has fostered the emergence of personalized disciplines. It is important to recognize that diverse populations often manifest unique pathogenic mechanisms and distinct clinical prognostic characteristics (Wu et al., 2022). Likewise, subgroups within individual samples are characterized by different functional gene sets, including immune-related and metabolism-related genes (Karim et al., 2021). Unsupervised consensus clustering using hedgehog-related genes revealed the presence of two subtypes. GSVA and ssGSEA indicated higher hedgehog activity in subtype 1, with immune differences observed between the two subtypes. Most hedgehog-related genes were significantly upregulated in subtype 1. FGSEA and GSVA showed enrichment of hypoxia, apoptosis, TNF signaling *via* NF-kB, angiogenesis, inflammatory response, and extracellular matrix processes in subtype 1. This further supports the notion that hedgehog exacerbates the development of OA. Additionally, based on the key modules identified by WGCNA, we also found associations between the hedgehog subtype, extracellular matrix, and inflammation-related pathways. Our study suggests that hedgehog-related genes play an important role in regulating the extracellular matrix and inflammatory responses, contributing to the pathogenesis of OA. Furthermore, using the Cmap database, we analyzed drugs that could reverse high hedgehog activity and identified some potentially applicable drugs, thus expanding the therapeutic options for treating OA. There are already some drugs that have shown potential as treatment options for osteoarthritis (OA). Palmatine (Pal) has been found to have no impact on chondrocyte viability, reduce the expression of matrix metalloproteinases (MMPs), and enhance the inhibitory effect of the Hedgehog signaling pathway inhibitor (cyclopamine) (Zhou et al., 2016). Low dose of indomethacin and Hedgehog signaling inhibitor administration synergistically attenuates cartilage damage in osteoarthritis by controlling chondrocytes pyroptosis (Liu et al., 2019). The kappa opioid receptor acts as a potential therapeutic target for OA by inhibiting the Hedgehog signaling pathway (Weber et al., 2020). Personalized treatment utilizes molecular diagnostics and genetic testing to accurately determine the type, subtype, and risk factors of diseases. This aids in early disease detection, early intervention, and the development of more appropriate treatment plans for patients. By detecting specific changes in the hedgehog pathway, we may be able to identify OA patients who are most likely to benefit from hedgehog-targeted therapy. Our study only provides a potential direction for personalized treatment in OA patients. Due to significant individual variations, the specific mechanisms and value of Hedgehog subtypes in OA patients remain unclear, and therefore, further experimental and clinical research is needed. Additionally, large-scale experiments and clinical studies based on disease progression or other assessment systems, such as pain severity, degree of joint function impairment, and cartilage degeneration, are still required to validate the differences between the two subtypes.

To further validate and explore our findings, we analyzed scRNA-seq data to describe the cellular heterogeneity of the hedgehog signaling pathway at the single-cell level. We found significant differences in hedgehog scores in chondrocyte clusters between the OA and normal groups, with higher hedgehog pathway activity observed in chondrocyte clusters from the OA group. We extracted chondrocyte clusters from the OA group for further analysis and conducted pseudotime analysis, which revealed expression changes in CSNK1G2, GAS1, GLI3, and WNT5A along the cellular differentiation trajectory. This indicates their involvement in the developmental processes of OA. Furthermore, through cell communication analysis, we discovered an association between chondrocytes with low hedgehog scores and macrophages, suggesting that the hedgehog pathway may act on macrophages to influence OA development. Studies have shown that agents such as cyclopamine (a Smoothened receptor inhibitor), GANT-58 (a GLI1 inhibitor), or GANT-61 (a GLI1/2 inhibitor) significantly inhibit RANKL-induced differentiation of bone marrow macrophages (Kohara et al., 2020). Macrophages in the tumor microenvironment (TME) play a critical role in tumor growth by influencing HH signaling pathways (Zhang et al., 2021). Hedgehog activity regulates the metabolism and bioenergetic programs of tumor-associated macrophages, promoting their immunosuppressive polarization (Hinshaw et al., 2021). Additionally, we explored potential ligand-receptor interactions, including ANGPTL2-PIRB, ANGPTL4-SDC2, CSF1-CSF1R, and IL6-(IL6R + IL6ST). Hedgehog activation can greatly enhance paracrine interactions between macrophages, progenitor cells, and endothelial cells through the Csf1 signaling pathway (Zhao et al., 2020). IL-6, mIL-8, Mcp-1, and M-csf (Csf1) are direct target genes of GLI1 and are involved in the recruitment of activated fibroblasts and immune cells in the pancreas (Mathew et al., 2014). Overall, our study provides in-depth insights into the complex connection between the hedgehog pathway and macrophages as important factors in the progression of OA. These findings suggest that hedgehog may participate in OA development through its effects on macrophages.

Indeed, our study has certain limitations that should be acknowledged. Firstly, despite our efforts to include multiple datasets related to OA, it would be beneficial to incorporate a larger number of OA samples to validate the stability of the identified subtypes. Secondly, rigorous clinical validation is required to establish the specificity and effectiveness of hedgehog subtypes. Additionally, further investigation is warranted to explore the potential correlations between hedgehog genes, ECM, and macrophages. Lastly, elucidating the role of hedgehog in OA through clinical sample and gene knockout experiments is a topic we plan to investigate in future studies.

In summary, our study combining Bulk RNA-seq and single-cell RNA-seq techniques discovered a significant increase in hedgehog pathway activity in OA and identified potential associations between hedgehog, ECM, and macrophages in the context of OA. The hedgehog correlation prediction model constructed using 10 machine learning algorithms exhibited promising diagnostic value. Our research provides valuable insights into the underlying mechanisms of OA involving hedgehog signaling and offers guidance for drug screening, personalized treatment approaches, and immunotherapy strategies for individuals with OA.

## Data Availability

The original contributions presented in the study are included in the article/Supplementary Material, further inquiries can be directed to the corresponding authors.
