# Supplementary figures and images for: Characterizing hedgehog pathway features in senescence associated osteoarthritis through Integrative multi-omics and machine learning analysis
Source: Front Genet. 2024 Feb 20;15:1255455. doi: 10.3389/fgene.2024.1255455 (PMC10912584; doi:10.3389/fgene.2024.1255455)

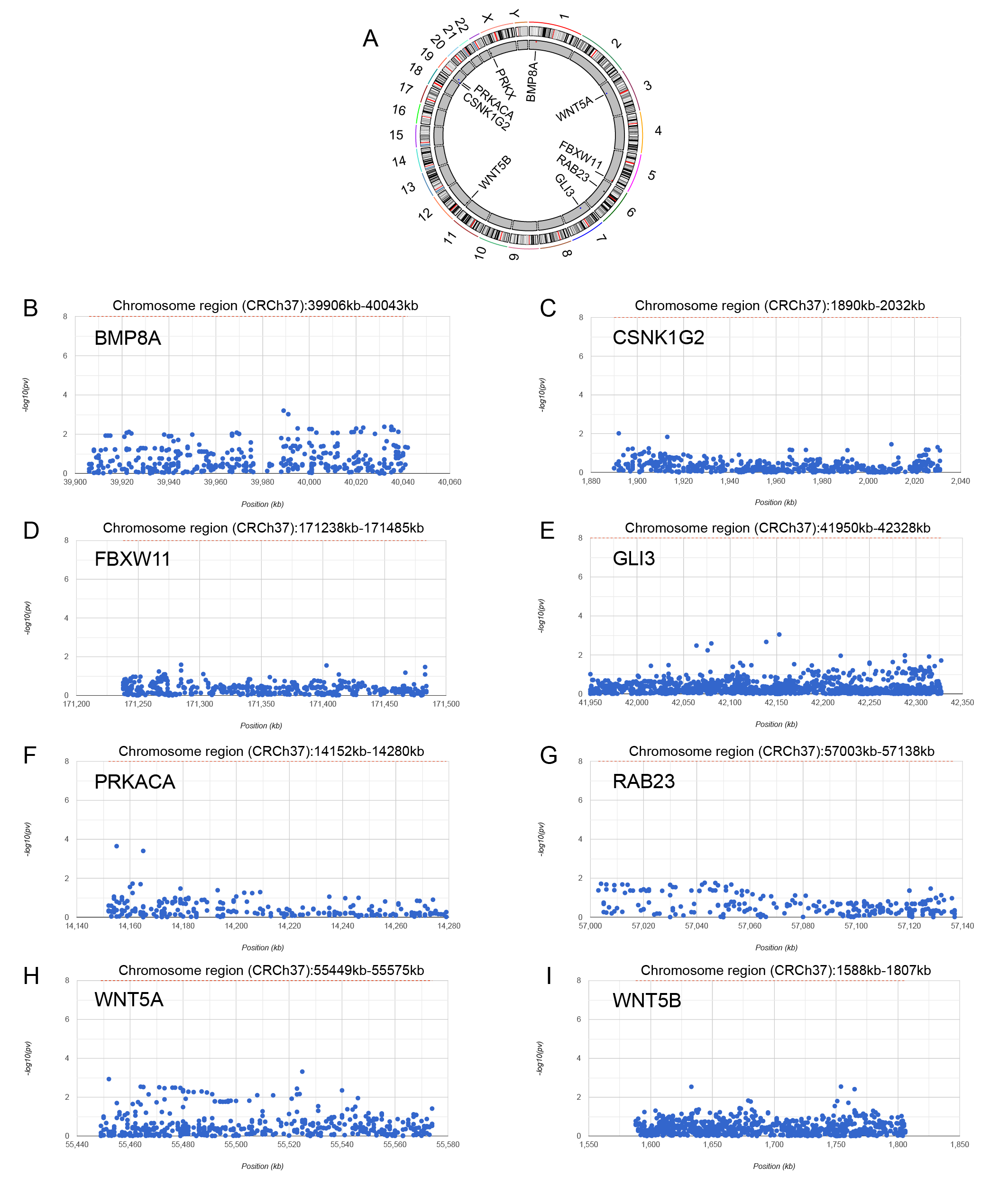

Supplement: Supplementary file 1 [file Image1.TIF]
